# DFA-YOLO: an enhanced YOLOv11-OBB and knowledge distillation-based maize stomata detection system

**DOI:** 10.3389/fpls.2026.1852592

**Published:** 2026-06-26

**Authors:** Zhenzhen Lin, Rui Liu, Antong Deng, Zhongxiang Xing, Huajuan Gao, Ziqi Yang, Yanxi Liu, Yuling Liu, Zhuolun He, Binhong Zhou, Jie Xu, Yan Guo, Zhiyong Li

**Affiliations:** 1 College of Information Engineering, Sichuan Agricultural University, Ya’an, Sichuan, China; 2 College of Mechanical and Electrical Engineering, Sichuan Agricultural, University, Ya’ an, Sichuan, China; 3 Maize Research Institute, Sichuan Agricultural University, Wenjiang, Sichuan, China; 4 State Key Laboratory of Crop Gene Exploration and Utilization in Southwest China, Sichuan Agricultural University, Wenjiang, Sichuan, China

**Keywords:** detection platform, focaler-CIoU, knowledge distillation, stomatal detection, YOLOv11-OBB

## Abstract

**Introduction:**

Stomata are vital gatekeepers of plants that regulate the fundamental trade-off between carbon gain and water loss. Precise, high-throughput identification of stomatal traits is therefore essential for assessing plant stress tolerance and water-use efficiency. However, conventional bounding box detection struggles to accurately localize densely distributed and arbitrarily oriented stomata.

**Methods:**

This study proposes DFA-YOLO, an enhanced YOLOv11-OBB (Oriented Bounding Box) model for orientation-aware maize stomatal localization and preliminary OBB-derived trait extraction. Based on a maize stomatal dataset expanded from 1,053 to 3,597 microscopic images, DFA-YOLO integrates three task-specific components: (1) a cross-dataset MGD distillation strategy that transfers structural priors from single-stomata images to dense multi-stomata scenes; (2) a fixed-threshold Focaler-CIoU localization-loss reweighting strategy (u_stomata_ = 0.95, d_stomata_ = 0.00) to emphasize hard positive samples during OBB regression; and (3) a C3k2_AssemFormer feature-aggregation module that combines convolutional local feature extraction with linear attention-based context aggregation.

**Results:**

DFA-YOLO achieved 94.1% mAP50, 84.8% mAP75, 74.1% mAP50-95, and 90.0% recall, with higher recall, mAP50, mAP75, and mAP50-95 than the YOLOv11-OBB baseline under the same OBB evaluation protocol. When deployed on an automated platform, the system processed images at 44.9 FPS and supported stomatal localization, density estimation, and preliminary orientation-aware size description.

**Discussion:**

Under the tested maize microscopic imaging workflow, DFA-YOLO enables rapid extraction of detection-oriented stomatal traits and provides a prototype tool for high-throughput maize stomatal phenotyping.

## 1 Introduction

As a vital link between plant genotypes and phenotypes, plant phenomics has become a cutting-edge area of contemporary agricultural research (Jin et al., 2025). In this context, the density, morphology, and dynamics of stomata—the primary channels for gas and water exchange between plants and the atmosphere—directly affect photosynthetic capacity and water-use efficiency (Carminati et al., 2020; Cernusak and De Kauwe, 2021; Buckley, 2023). Research has shown that stomatal opening and closure are regulated by multiple environmental and physiological factors, including water availability (Carminati et al., 2020; Ghannoum et al., 2025), light intensity (Cernusak and De Kauwe, 2021), CO₂ concentration (Buckley, 2023), and guard cell wall properties (Amsbury et al., 2016). Given this multifactorial regulation, accurate quantification of stomatal traits is essential for understanding plant physiological and ecological processes.

Despite the importance of stomatal phenotyping, traditional analysis methods primarily rely on manual microscopic counting (Ha et al., 2021) or semi-automated processing workflows using ImageJ. These approaches are not only labor-intensive and limited by low throughput, but also suffer from high inter-observer variability, severely hindering the development of large-scale plant phenotyping. Demonstrating the potential of deep learning in this domain, LabelStoma developed a stomatal detection tool based on the YOLO framework (Casado-García et al., 2020). Subsequent studies, such as I3-YOLOv8s, have further improved detection accuracy for irregularly shaped stomata (Gong et al., 2025). However, most of these methods employ horizontal bounding boxes (HBB), which struggle to accurately enclose stomata with arbitrary orientations in natural samples. Consequently, this leads to excessive background inclusion and suboptimal localization accuracy.

In addition to bounding-box-based stomatal detectors, several domain-specific stomatal phenotyping tools have been developed for automated stomatal identification, counting, and morphometric analysis. StomataCounter introduced a convolutional-neural-network-based system for automatic stomatal identification and counting from microscopic images (Fetter et al., 2019). DeepStomata combined stomatal region detection with pore isolation by image segmentation for automated aperture measurement (Toda et al., 2018). StomaAI provided an efficient deep-computer-vision tool for measuring stomatal pores and density (Sai et al., 2023). More recently, StoManager1 integrated convolutional neural networks with empirical and geometrical algorithms to detect, count, and measure multiple stomatal and guard-cell traits (Wang et al., 2024), while LeafNet enabled stomatal localization, pavement-cell segmentation, and extraction of multiple epidermal morphological parameters (Li et al., 2022a). These segmentation-based or hybrid detection–segmentation systems are biologically important because they can directly represent stomatal boundaries, pore contours, aperture geometry, guard-cell morphology, and epidermal cell traits.

However, full pixel-level segmentation generally requires more labor-intensive annotations than bounding-box-based detection, especially for dense microscopic fields containing numerous stomata. In the present study, OBB detection is positioned as a practical compromise for high-throughput maize stomatal localization when pixel-level masks are costly or unavailable. Compared with horizontal bounding boxes, OBBs better encode stomatal long-axis orientation and reduce unnecessary background inclusion. Compared with segmentation masks, OBBs cannot capture pore contours or guard-cell boundaries, but they can provide efficient estimates of stomatal position, density, long-axis orientation, and coarse size descriptors in dense maize stomatal images. In this study, OBB-derived size descriptors are used as coarse geometric proxies for stomatal extent, whereas aperture geometry, pore area, and guard-cell morphology remain segmentation-level traits.

Oriented object detection provides an orientation-aware representation for objects whose long axes are not aligned with image axes. This representation is relevant to stomatal localization because maize stomata in microscopic images are elongated, densely distributed, and often arbitrarily oriented. However, stomatal microscopy differs from remote sensing object detection: stomata are small biological structures with blurred boundaries and morphometric traits that cannot be fully represented by rotated rectangles. Therefore, OBB detection is used here as a task-specific localization strategy for estimating stomatal position, density, long-axis orientation, and coarse size descriptors, rather than as a general solution for stomatal morphology analysis. Existing OBB detectors, including R3Det (Yujie et al., 2022), S2A-Net (Li et al., 2023), RQFormer (Zhao et al., 2025), RO-DETR (Dang et al., 2025), and OriMamba (Xiao et al., 2025), provide technical references for orientation-aware localization, while TSAF-Net (Chen et al., 2024) demonstrates the use of oriented detection in plant phenotyping. These studies motivate orientation-aware detection, but dense stomatal microscopy still requires task-specific adaptation.

Within this OBB-based localization setting, several practical issues still need to be addressed. First, transfer from isolated single-stomata images to dense multi-stomata fields is nontrivial, because dense fields contain many adjacent stomata and require more labor-intensive OBB annotations (Casado-García et al., 2020; Gong et al., 2025). Second, hard instances with dense adjacency, blurred boundaries, or partial occlusion can reduce count coverage and OBB localization quality when optimization is dominated by easier samples (Lin et al., 2020; Li et al., 2022b; Zhang and Zhang, 2024). Third, dense stomatal images require joint modeling of local boundary cues and broader spatial context. These considerations define the scope of the present detection-oriented framework, whereas detailed pore contours, guard-cell boundaries, and fine-scale shape traits remain better addressed by segmentation-based morphometric analysis.

To address the above challenges under an OBB-based detection setting, this study proposes DFA-YOLO, an enhanced YOLOv11-OBB framework specifically designed for dense maize stomatal localization. This work focuses on orientation-aware detection and preliminary OBB-derived trait extraction under conditions where pixel-level annotations are costly or unavailable. The main contributions are summarized as follows:

1. A maize stomatal oriented detection dataset was constructed, covering both isolated single-stomata images and dense multi-stomata microscopic scenes, thereby providing the data basis for cross-dataset knowledge transfer.
2. A cross-dataset knowledge distillation strategy based on Mask-based Global Distillation (MGD) was adapted to follow a “simple-to-complex” learning paradigm, transferring structural priors from isolated stomatal images to dense multi-stomata scenes to alleviate feature confusion, occlusion interference, and boundary ambiguity.
3. The Focaler-CIoU loss was adapted to the oriented detection framework as a fixed-threshold heuristic localization-loss reweighting strategy (u_stomata_=0.95, d_stomata_=0.00), which emphasizes difficult positive samples with blurred boundaries, dense adjacency, or partial occlusion during OBB regression.
4. The C3k2_AssemFormer module was integrated into the YOLOv11-OBB backbone to combine CNN-based local morphological feature extraction with efficient linear attention-based global aggregation, thereby enhancing the representation of multi-scale and arbitrarily oriented stomata.
5. A prototype stomatal detection and analysis platform was developed to support automated stomatal localization, density estimation, and preliminary orientation-aware trait extraction in maize microscopic images.

## 2 Materials and methods

### 2.1 Overview of the method

**Figure 1:**
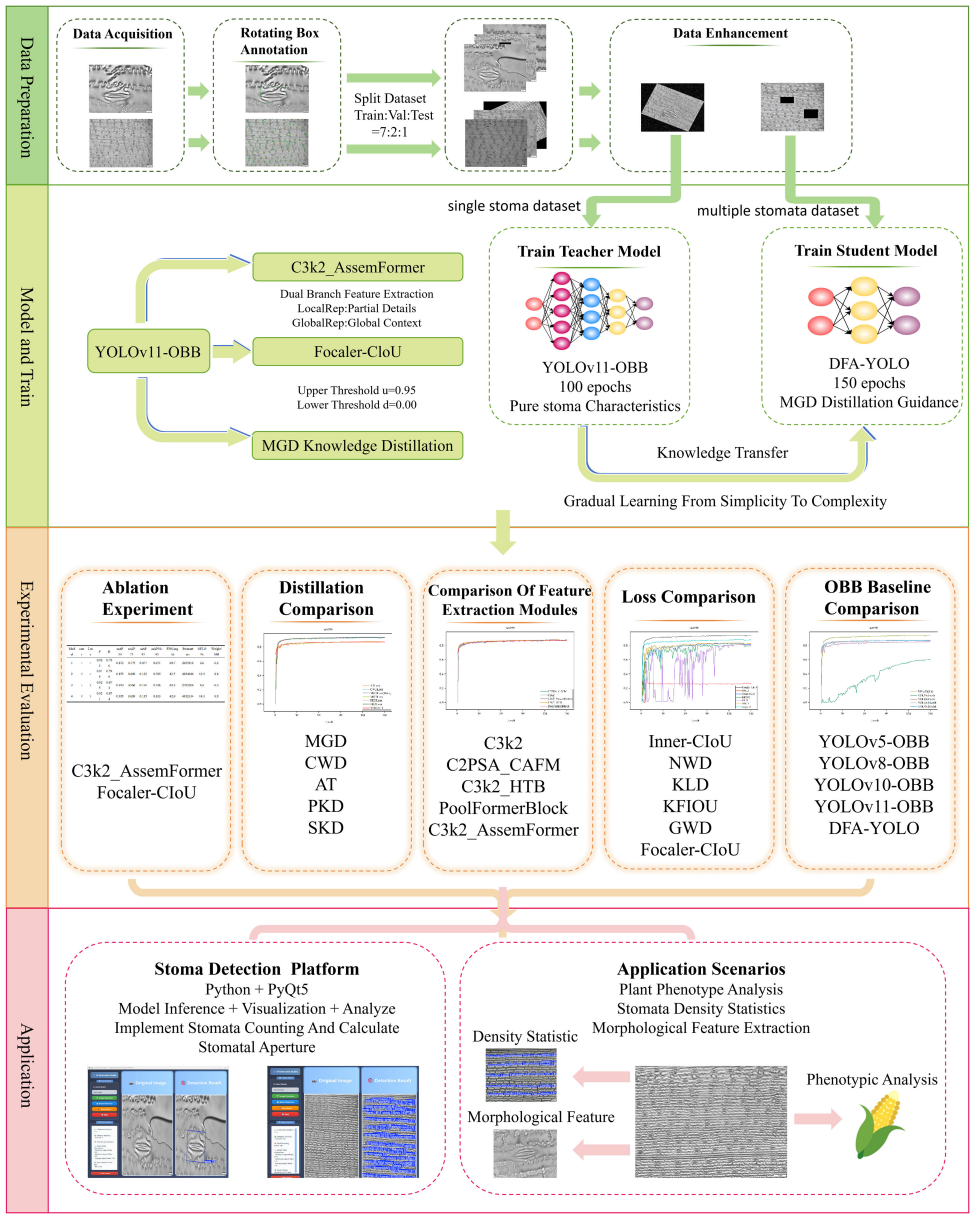
Overall experimental framework of the study. The workflow includes data preparation and augmentation, DFA-YOLO training with C3k2_AssemFormer, Focaler-CIoU, and MGD distillation, model evaluation, and GUI-based deployment for maize stomatal detection under the tested imaging workflow.

**Figure 1** illustrates the overall experimental framework of this study, including maize stomatal data acquisition, progressive model training, performance evaluation, and practical deployment.

### 2.2 Information gathering

Maize plants of the KN 5585 cultivar were grown under outdoor greenhouse conditions with routine water and fertilizer management and without drought, salinity, pathogen, or chemical stress treatments. Leaf samples were collected at the seedling stage when plants had reached the seven-leaf stage. Thirty plants were sampled, and all sampled plants belonged to the same cultivar and growth stage to reduce variation caused by genotype and developmental stage.

To remove surface dust, oils, and loose particles, the abaxial surface of maize leaves was gently cleaned with soft tissue. A thin, uniform layer of transparent nail polish was then applied to approximately 1 cm² of the abaxial epidermis. The nail polish was allowed to air-dry for 3–8 min. To ensure complete adhesion, transparent adhesive tape was carefully placed onto the dried nail polish surface and gently pressed with a finger or rubber eraser.

The tape was then carefully peeled off by lifting one corner using fine-tipped forceps. The separated tape, bearing the epidermal impression, was carefully mounted onto a microscope slide to complete the preparation.

All stomatal images were acquired using an Olympus BX53 microscope (Olympus Corporation, Tokyo, Japan) equipped with an Olympus DP74 digital camera. Different objective configurations were used according to the imaging task. Single-stomata images were acquired using a 100× oil-immersion objective (NA 1.25). Pixel calibration was performed using a stage micrometer, and the calibrated spatial resolution was 0.10 μm per pixel.

Multi-stomata images were acquired using a 20× objective (NA 0.45) and a 40× objective (NA 0.60). Images were captured at a resolution of 4104 × 3096 pixels. Pixel calibration was also performed using a stage micrometer. The calibrated horizontal field-of-view widths were approximately 900 μm for the 20× objective and 450 μm for the 40× objective, corresponding to horizontal spatial resolutions of approximately 0.219 μm per pixel and 0.110 μm per pixel, respectively.

Images were captured in bright-field imaging mode using a halogen light source. During acquisition, the illumination intensity was fixed at 70%, the exposure time was set to 100 ms, and the camera gain was set to 100. To improve imaging consistency and experimental reproducibility, all samples were imaged on the same day by the same operator. Within each imaging protocol, microscope and camera settings were kept constant throughout image acquisition, and unified focusing and field-of-view selection criteria were used to minimize operator-dependent variation.

A total of 551 single-stomata images and 502 multi-stomata images were obtained from 30 plants.

### 2.3 Information processing

All images in the dataset were manually annotated using oriented bounding boxes (OBB) with the LabelImg-OBB tool. Each stomatal instance was defined by five parameters: center coordinates (x, y), width and height (w, h), and rotation angle θ ∈ [-180°, 180°].

A differentiated data augmentation strategy was employed to enhance model robustness. For single-stomata images, nine augmentation techniques were applied: rotation (± 30°), horizontal and vertical flipping, brightness/contrast adjustment (0.7-1.3), HSV color jittering, Gaussian, speckle, and salt-and-pepper noise, Gaussian, median, and bilateral blur, Cutout (DeVries and Taylor, 2017), and affine transformation. Two random augmentations were applied to each image. For multi-stomata images, six conservative augmentation techniques were used: rotation (± 15°), flipping, Gaussian noise, Gaussian blur, brightness/contrast adjustment (0.8-1.2), and Cutout (DeVries and Taylor, 2017). To preserve the spatial relationships among stomata, only one random augmentation was applied to each image.

**Figure 2:**
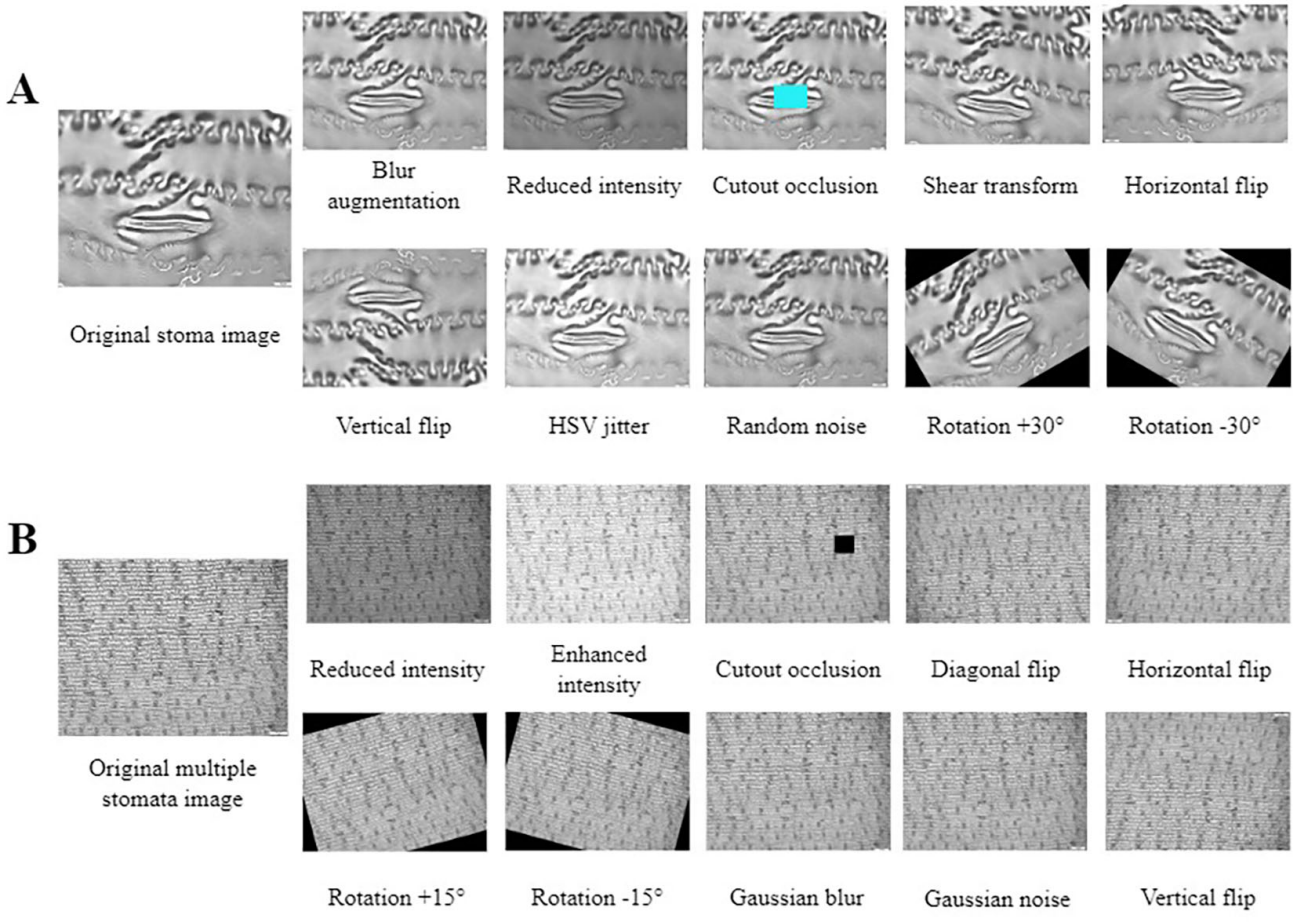
Representative samples from the original and augmented datasets. Examples of augmentation for single-stomata (top row) and multi-stomata (bottom row) images. **(A)** To enhance morphological diversity, single-stomata images were subjected to nine augmentation techniques: rotation (± 30°), horizontal/vertical flipping, brightness/contrast adjustment, HSV jittering, noise addition (Gaussian, speckle, salt-and-pepper), blurring (Gaussian, median, bilateral), Cutout, and affine transformation. **(B)** To preserve spatial relationships among stomata, multi-stomata images were subjected to six conservative augmentations: rotation (± 15°), flipping, brightness/contrast adjustment, Gaussian noise, Gaussian blur, and Cutout.

Original images were first grouped by plant identity and then split at the plant level into training, validation, and test sets at an approximate ratio of 7:2:1 before augmentation. All images from the same plant were assigned to the same subset to avoid plant-level information leakage between training and evaluation data. Data augmentation was applied only to the plant-wise training set, while validation and test images remained original and non-augmented. After training-set augmentation, the dataset contained 3, 597 images, with all bounding box annotations updated accordingly. Dataset statistics are provided in **Supplementary Table 1**, and representative original and augmented training images are shown in **Figure 2**.

### 2.4 Architecture of network

Three task-specific adaptations were incorporated into the YOLOv11-OBB architecture: (1) a cross-dataset knowledge distillation strategy employing a progressive learning paradigm from simple to complex scenarios (single-stomata to multi-stomata), (2) a fixed-threshold Focaler-CIoU localization-loss reweighting strategy for difficult positive samples, and (3) the C3k2_AssemFormer module, which integrated CNN-based local feature extraction with linear attention-based global aggregation.

**Figure 3:**
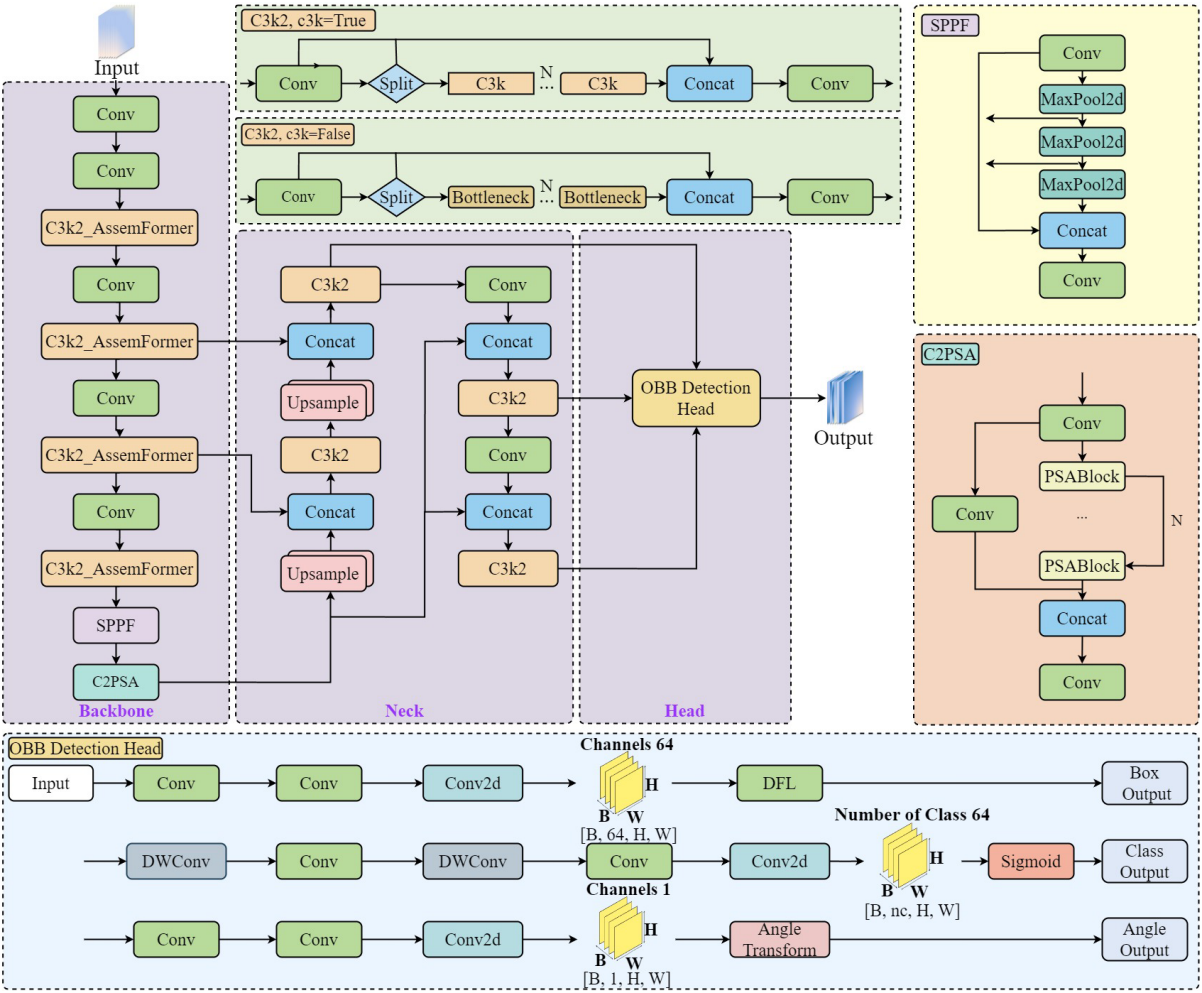
DFA-YOLO network architecture. The architecture comprised three components: Backbone (C3k2_AssemFormer modules integrated with SPPF and C2PSA for multi-scale feature extraction), Neck (C3k2-based dual-path structure with bidirectional feature pyramid network (FPN-PAN) for feature fusion), and OBB Detection Head (employing Distribution Focal Loss (DFL), depthwise separable convolutions, and angle regression for bounding box regression, classification, and rotation angle prediction). Right panel: detailed architectures of SPPF (Spatial Pyramid Pooling - Fast) and C2PSA (Cross-Stage Partial with Spatial Attention) modules. Feature dimensions are denoted as [B, C, H, W], representing batch size, channels, height, and width.

DFA-YOLO adopted a Backbone–Neck–Head architecture, in which the backbone, neck, and head were responsible for multi-scale feature extraction, feature pyramid fusion, and oriented bounding box regression, respectively (**Figure 3**).

#### 2.4.1 C3k2_AssemFormer module

Dense maize stomatal images require both local boundary representation and global spatial context. To address this requirement, C3k2_AssemFormer was adapted from the AssembleFormer architecture and integrated into the YOLOv11-OBB backbone. The module uses a dual-branch design: LocalRep extracts local morphological features through convolutional operations, whereas GlobalRep aggregates broader spatial context using linear attention. This design supports OBB localization in dense stomatal images while avoiding the computational cost of full self-attention.

Given an input feature map F_in_∈R^B×C×H×W^, C3k2_AssemFormer first generates local features through the LocalRep branch, as shown in Equation 1:

(1) $$F_{l}=LocalRep(F_{in}),F_{l}\in R^{B\times d\times H\times W},d=C/2$$

The local feature F_l_ is then converted into patch tokens before global context aggregation, as shown in Equation 2:

(2) $$Z=Pack\left(F_{l}\right)$$

where Z∈R^d×N^ denotes the packed patch-token representation and N is the number of patch tokens. The GlobalRep branch applies linear self-attention to Z and reshapes the output back to the spatial feature map, as shown in Equation 3:

(3) $$F_{g}=Unpack\left(LSA\left(Z\right)\right),F_{g}\in R^{B\times d\times H\times W}$$

Within the linear self-attention block, a channel-wise global context vector is first computed, as shown in Equation 4:

(4) $$C_{g}={\left[Soft\max\left(q\right)K^{T}\right]}^{T}$$

and the attention output is then obtained by modulating the value features, as shown in Equation 5:

(5) $$LSA\left(Z\right)=V⨀C_{g}$$

The query, key, and value features were projected from the packed token representation Z. Here, q∈R^1×N^ is the global query vector, K∈R^d×N^ denotes key features, V∈R^d×N^ denotes value features, C_g_ denotes the channel-wise global context vector, and ⨀ denotes element-wise multiplication with broadcasting along the token dimension. This operation aggregates global context without pairwise token interaction, reducing the computational complexity from O(N^2^) to O(N).

The local and global features were fused by channel-wise concatenation and 1×1 convolutional projection, as shown in Equation 6:

(6) $$F_{f}=Conv\text{Pr}oj\left(\left[F_{l},F_{g}\right]\right)$$

where [F_l_, F_g_] denotes channel-wise concatenation. The final module output was obtained through residual connection and stochastic-depth regularization, as shown in Equation 7:

(7) $$F_{out}=F_{in}+DropPath_{p}\left(F_{f}\right)$$

During training, DropPath_p_(·) randomly drops the fused branch with probability p and rescales the retained branch, as shown in Equation 8:

(8) $$DropPath_{p}\left(X\right)=\frac{m}{1-p}X,m\sim Bernoulli\left(1-p\right),m\in {\left\{0,1\right\}}^{B\times 1\times 1\times 1}$$

During inference, DropPath_p_(X)=X. In this study, p=0.1. The residual connection preserves the input pathway, while stochastic depth regularizes the AssemFormer branch to reduce overfitting risk.

**Figure 4:**
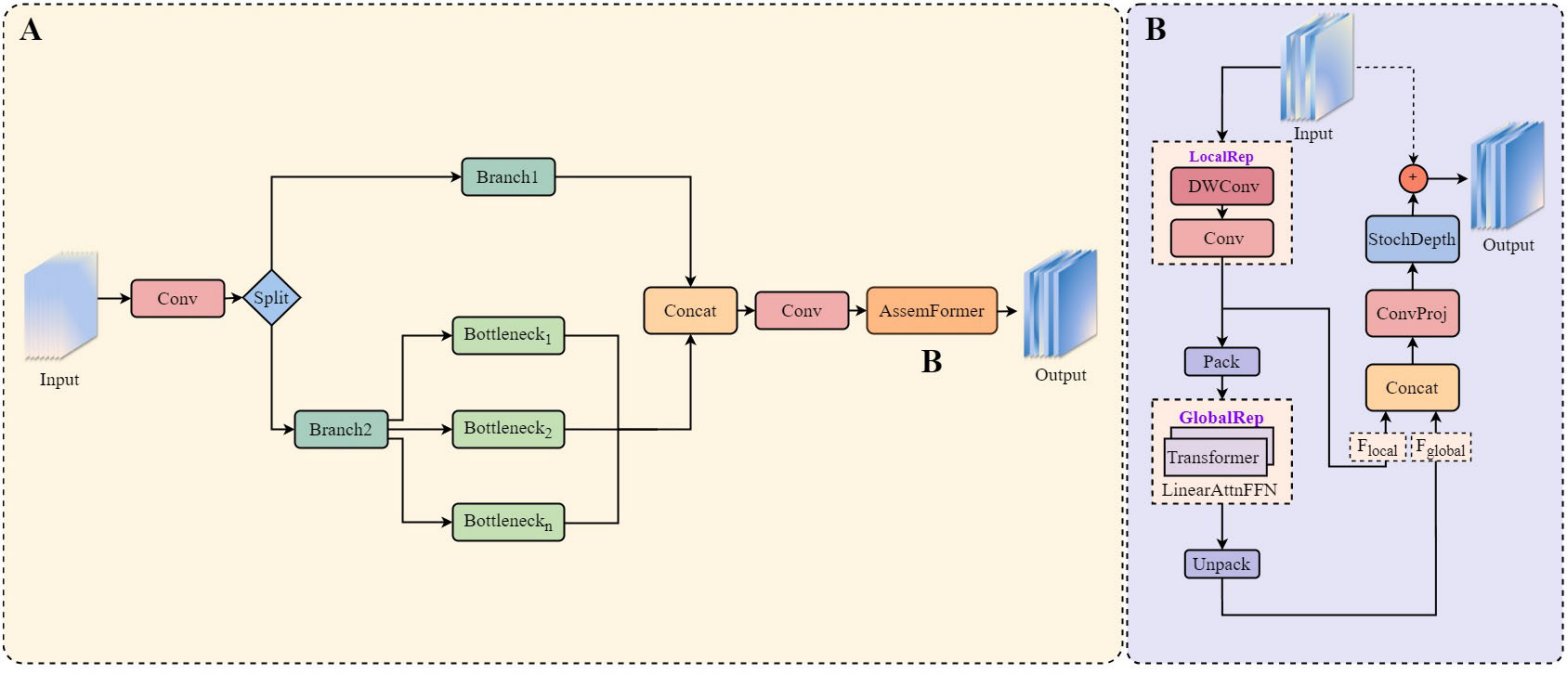
C3k2_AssemFormer architecture. **(A)** Branch 1 provides direct feature propagation, while Branch 2 processes features through n cascaded Bottleneck blocks, concatenation, and convolutional refinement before feeding them into AssemFormer. **(B)** AssemFormer integrates local and global representations. LocalRep uses depthwise separable convolutions for local feature extraction, whereas GlobalRep uses linear self-attention and feed-forward networks for global context aggregation. ConvProj denotes the 1×1 convolutional projection after local–global feature concatenation, and StochDepth denotes the DropPath-based stochastic-depth operation.

C3k2_AssemFormer was integrated into the standard C3k2 structure and deployed at Backbone layers 2, 4, 6, and 8. Shallow layers captured local boundary and morphology-related features, whereas deeper layers aggregated broader spatial context for dense stomatal localization. Through the Neck, shallow local features and deep contextual features were fused for final OBB regression. The architecture of the module is shown in **Figure 4**.

#### 2.4.2 Focaler-CIoU loss

Sample difficulty exhibited clear stratification in maize stomatal detection. In single-stomata scenarios, isolated stomata with clear boundaries were easy to detect, while in multi-stomata scenarios, samples with blurred boundaries or partial occlusion were challenging to detect. Extremely hard samples included false positives from background regions. The standard CIoU loss applied uniform gradients to all samples without differentiating optimization based on sample difficulty. This resulted in insufficient optimization for hard samples, wasted computational resources on easy samples, and susceptibility to outlier interference during late-stage training.

**Figure 5:**
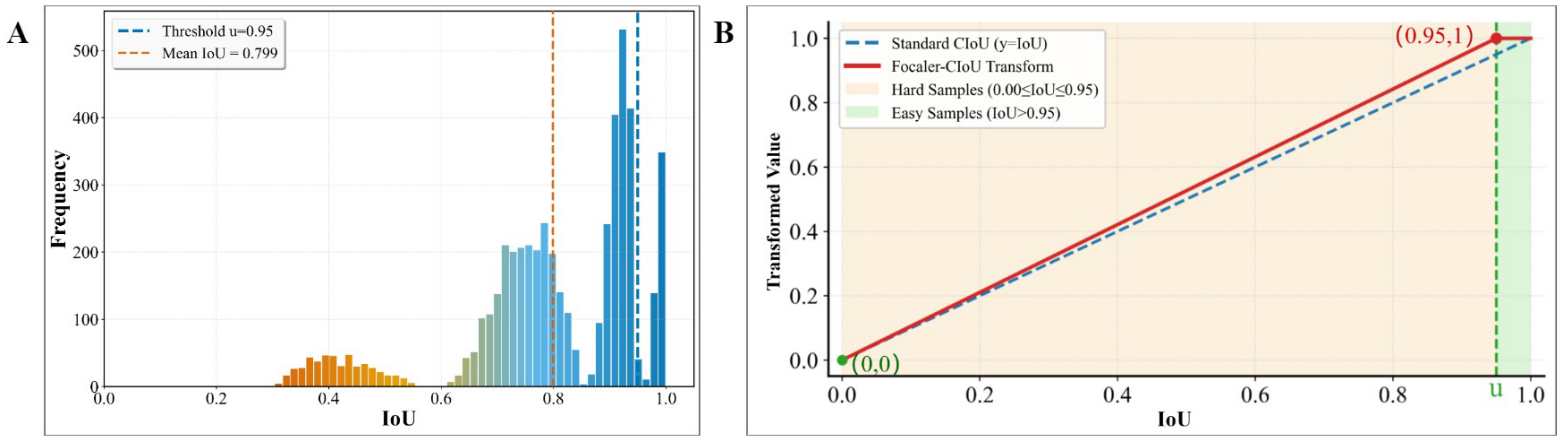
Focaler-CIoU loss transformation mechanism and fixed-threshold setting. **(A)** Training-sample IoU distribution shown as a descriptive reference for the localization-overlap range observed during training. Blue dashed line: fixed upper threshold u_stomata_=0.95. **(B)** Transformation curves: standard CIoU (blue dashed) vs. Focaler-CIoU (red solid). For IoU_stomata_<0.95, linear scaling maintained localization-loss optimization; for IoU_stomata_>0.95, mapping to 1.0 reduced further IoU-loss optimization.

Focaler-CIoU was adapted to the stomatal OBB detection task as a fixed-threshold heuristic localization-loss reweighting strategy. In this study, u_stomata_=0.95 and d_stomata_=0.00 were treated as fixed hyperparameters rather than trainable loss parameters or statistically optimized thresholds, and these values remained unchanged during model optimization. To examine whether the result depended on a single upper-threshold setting, a sensitivity analysis was conducted by fixing d_stomata_=0.00 and evaluating neighboring u_stomata_ values of 0.900, 0.925, 0.950, and 0.975. As shown in **Figure 5**, predictions with IoU_stomata_>0.95 were mapped to 1.0, whereas predictions with 0≤IoU_stomata_ ≤ 0.95 were linearly scaled by IoU_stomata_/0.95. This reweighting emphasized difficult positive samples with blurred boundaries or partial occlusion while avoiding additional angle-specific loss terms.

The Focaler-CIoU loss function was built upon the standard CIoU loss. First, the base IoU_stomata_ between predicted and ground truth stomatal bounding boxes was computed, as shown in Equation 9:

(9) $$L_{CIoU_{stomata}}=1-loU_{stomata}+\frac{\rho^{2}\left(b_{stomata},b_{gt\_stomata}\right)}{c^{2}}+\alpha_{stomata}\cdot v_{stomata}$$

where IoU_stomata_ denoted the intersection-over-union, c denoted the diagonal length of the smallest enclosing box, and ρ^2^(b_stomata_, b_gt_stomata_) denoted the squared Euclidean distance between the centers of the predicted and ground truth bounding boxes. α_stomata_ was the dynamic weighting coefficient, while v_stomata_ measured the aspect-ratio consistency.

Building on this, the Focaler transformation mechanism partitioned samples into two categories based on IoU_stomata_ values. For IoU_stomata_>0.95, the transformed value was mapped to 1.0 to reduce further IoU-loss optimization of already well-aligned predictions. For 0≤IoU_stomata_ ≤ 0.95, the transformed value was linearly scaled by IoU_stomata_/0.95 to maintain optimization pressure on positive samples requiring further localization refinement. This mapping strategy enforced strict criteria for high-quality predictions (IoU_stomata_ > 0.95) while ensuring continuous optimization for most samples (IoUstomata ≤ 0.95). Mathematically, this transformation was formulated as shown in Equation 10:

(10) $$Focaler_{stomata}\left(IoU_{stomata}\right)=\{\begin{array}{ll} 1.0, & \text{if }IoU_{stomata}>0.95\\ \frac{IoU_{stomata}}{0.95}, & \text{if }0\leq IoU_{stomata}\leq 0.95 \end{array}$$

In this work, the upper and lower thresholds were set to u_stomata_=0.95 and d_stomata_=0.00, respectively. These values remained fixed during model optimization and defined the piecewise Focaler transformation used for localization-loss reweighting. They were not treated as physical stomatal traits or as statistically optimized thresholds derived from the training-set IoU distribution. For each positive sample i, the target score weight w_i_stomata_ was inherited from the YOLO label-assignment process and used only as the sample weight for loss aggregation. For the single-class stomatal detection task, it can be written as shown in Equation 11:

(11) $$w_{i_{stomata}}=s_{i_{stomata}}^{target}$$

where $s_{i_{stomata}}^{target}$ denotes the assigned target score of positive sample i. The final Focaler-CIoU loss was then computed as a target-score-weighted average over all positive samples, as shown in Equation 12:

(12) $$L_{Focaler-CIoU_{stomata}}=\frac{\sum_{i=1}^{N_{pos\_stomata}}\omega_{i_{stomata}}\left[1-Focaler_{stomata}\left(IoU_{i_{stomata}}\right)\right]}{\sum_{i=1}^{N_{pos\_stomata}}\omega_{i_{stomata}}+\epsilon }$$

where N_pos_stomata_ denotes the number of positive samples, w_i_stomata_ denotes the target score weight assigned to positive sample i, and ϵ is a small constant used to avoid division by zero. Unlike axis-aligned bounding boxes, the method employed 5-dimensional oriented bounding boxes (x, y, w, h, θ) via the FocalerCIoURotatedBboxLoss module. Spatial IoU_stomata_ was computed from (x, y, w, h), while θ enabled orientation regression. Additionally, Distribution Focal Loss (DFL) was integrated to model coordinates as distributions rather than point estimates, enhancing localization accuracy.

This fixed-threshold design was used as a heuristic localization-loss reweighting strategy rather than a theoretically new IoU loss. The lower threshold preserved optimization pressure for low-overlap positive samples, whereas the upper threshold reduced unnecessary IoU-loss optimization for already well-aligned predictions. Thus, Focaler-CIoU prioritized difficult positive samples during bounding-box regression without changing the YOLO label-assignment process.

#### 2.4.3 Knowledge distillation

**Figure 6:**
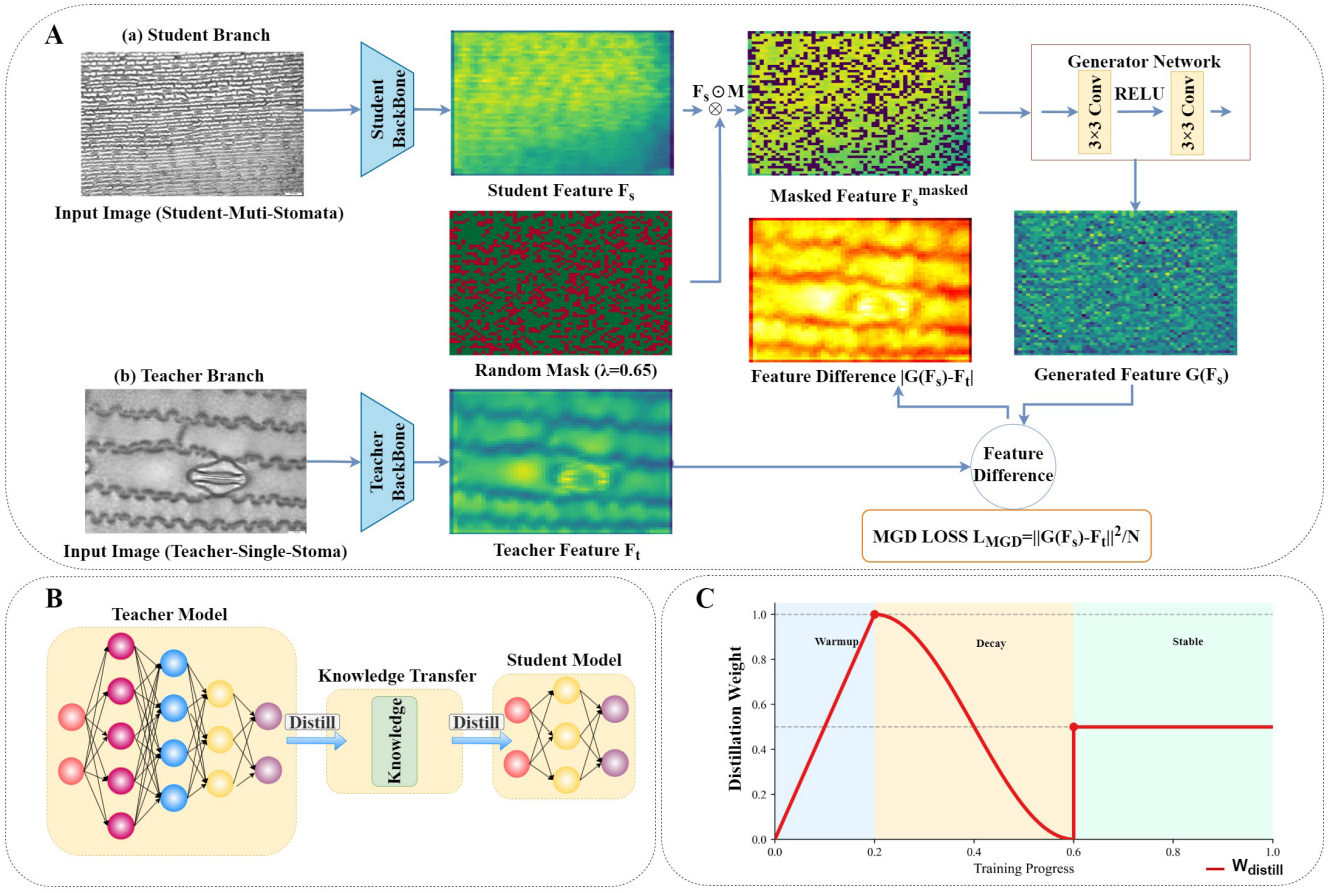
Framework for knowledge distillation. **(A)** MGD masking mechanism: Teacher extracted F_t_ from single-stomata images; student processed multi-stomata images to generate F_s_, transformed via generator (3×3 conv + ReLU) and mask (λ=0.65) to G(F_s_). MGD loss ||G(F_s_)-F_t_||² transferred structural knowledge. **(B)** Teacher-student knowledge distillation process. **(C)** Distillation weight scheduling: warm-up (0.0→1.0), decay (1.0→0.5), stabilization (0.5).

Single-stomata datasets were suitable for learning intrinsic stomatal features such as kidney-shaped guard cell contours and pore geometry, because each image contained one or two isolated stomata. In contrast, multi-stomata datasets contained approximately 170 stomata per image on average, where occlusion and dense arrangements complicated detection. Although directly pooling single-stomata and multi-stomata images for mixed-complexity training is a possible alternative, this strategy would treat isolated clean images and dense occluded images as samples from the same target distribution. In this study, the target deployment domain was dense multi-stomata microscopy. Therefore, the single-stomata dataset was used as an auxiliary source domain for learning structural priors, whereas the multi-stomata dataset was used as the target domain for student optimization. This design separated source-domain structural prior learning from target-domain dense-scene localization, allowing the student model to receive guidance from isolated stomatal morphology without treating single-stomata images as additional target-domain samples. The teacher model was trained for 100 epochs, while the student model was trained for 150 epochs. As shown in **Figure 6**, the distillation framework transferred knowledge from isolated stomatal images to dense multi-stomata scenes.

After comparing MGD with four alternative feature distillation methods (CWD, AT, PKD, and SKD), MGD was used to transfer structural priors from the single-stomata teacher to the dense multi-stomata student. Because the teacher and student processed images with different scene complexity, their feature distributions were not assumed to be identical. Therefore, the distillation objective was not exact feature matching, but weak structural guidance based on shared stomatal morphology, including guard-cell morphology, pore geometry, and boundary characteristics. The MGD mask (λ=0.65) randomly occluded student features and required the generator to reconstruct aligned features for comparison with teacher features. This encouraged the student network to learn structural representations transferable from isolated stomata to dense multi-stomata images, rather than simply memorizing teacher features. The MGD loss was defined as shown in Equation 13:

(13) $$L_{MGD_{stomata}}=\frac{1}{N_{stomata}}\times \sum {\left\|G_{i_{stomata}}\left(M⨀A_{i_{stomata}}\left(F_{s_{stomata}}\right)\right)-F_{t_{stomata}}\right\|}^{2}$$

where N_stomata_ was the number of feature maps, F_s_stomata_ and F_t_stomata_ were student and teacher features, M was the random mask, and G_i_stomata_ was the generator (3×3 convolution + ReLU). The generator served as a feature-alignment module that projected student features into a teacher-guided structural-prior space, enabling weak structural comparison despite differences in scene complexity. Distillation weight followed cosine annealing: warm-up to 1.0 (epochs 1-10), decay to 0.5 (epochs 11-60), stabilization at 0.5 (epochs 61-150). The scheduling function was defined as shown in Equation 14:

(14) $$w_{distill_{stomata}}\left(e\right)=w_{final_{stomata}}+0.5\times \left(w_{init_{stomata}}-w_{final_{stomata}}\right)\times \left[1+\cos\left(\pi \times \frac{e-e_{warm\;up_{stomata}}}{e_{decay_{stomata}}}\right)\right]$$

The parameters were w_init_stomata_=1.0, w_final_stomata_=0.5, e_warmup_stomata_=10, and e_decay_stomata_=50. Multi-scale features from Backbone layers 6, 8, and 10 were used for distillation, encoding both local stomatal details and global spatial context.

### 2.5 Metrics for evaluation

Model evaluation was based on the matching between predictions and ground truth, as defined in **Table 1**. The main experiments were conducted using the plant-wise 7:2:1 training, validation, and test split described in Section 2.3. To further evaluate the stability of the results beyond this fixed split, five-fold plant-wise cross-validation was conducted for the YOLOv11-OBB baseline and DFA-YOLO. In each fold, plants rather than individual images were used as the splitting unit, and data augmentation was applied only to the training fold. The cross-validation results were summarized as mean ± standard deviation across five folds. To test whether the mAP50–95 improvement over the baseline was statistically supported under identical plant-wise partitions, fold-wise mAP50–95 values of the two models were further compared using a paired two-sided Wilcoxon signed-rank test.

**Table 1:** Confusion matrix.

Confusion matrix		Prediction	
		Positive	Negative
Actual	Positive	TP	FN
	Negative	FP	TN

TP, True Positive; FN, False Negative; FP, False Positive; TN, True Negative.

Precision, Recall, mAP50, mAP75, mAP95, mAP50–95, FPS, Parameters, GFLOPs, and Weight are the measures used in this study to assess model performance.

In addition, to quantify the practical impact of the precision–recall trade-off in the YOLO-series OBB baseline comparison, diagnostic image-level statistics were calculated on the held-out maize multi-stomata test set. Using one-to-one OBB matching at IoU ≥ 0.5, the mean numbers of true positives, false positives, false negatives, predicted stomata, and count bias per image were summarized for each model. Because density estimation in the present workflow is count-based, count bias per image was used as a practical proxy for the effect on stomatal density estimates.

Precision: The percentage of true positives among all positive predictions, which indicates how well the model can filter out false positives, as defined in Equation 15.

(15) $$\text{Precision}=\frac{\text{TP}}{\text{TP}+\text{FP}}$$

Recall: The percentage of all true positive samples that are successfully identified, indicating how well the model can cover the target, as defined in Equation 16.

(16) $$\text{Recall}=\frac{\text{TP}}{\text{TP}+\text{FN}}$$

Average Precision (AP): Various Precision-Recall pairs can be obtained by varying the confidence threshold. The PR curve is created by joining these pairs. The area under this curve, or AP, has a value range of [0, 1], as defined in Equation 17.

(17) $$\text{AP}=\int_{0}^{1}\text{P}\left(\text{R}\right)\text{dR}$$

where recall rate R is the independent variable and P(R) is the precision function.

mAP50: The arithmetic mean of all category APs at an IoU threshold of 0.5 is known as mAP50 (mean Average Precision at IoU = 0.5), as defined in Equation 18. mAP50 is the same as AP50 for single-class detection tasks.

(18) $$\text{mAP}50=\frac{1}{\text{C}}\times \sum {\text{AP}}_{50}\text{(c)}$$

where C stands for the total number of categories.

mAP75 and mAP95: Similar to mAP50, mAP75 and mAP95 are the arithmetic means of all category APs calculated at IoU thresholds of 0.75 and 0.95, respectively. For single-class detection tasks, mAP75 and mAP95 are equivalent to AP75 and AP95.

mAP50–95: mAP50–95 is the mean Average Precision averaged over multiple IoU thresholds from 0.50 to 0.95 with a step size of 0.05, as defined in Equation 19.

(19) $$mAP50-95=\frac{1}{10C}\times \sum_{t\in \left\{0.50,0.55,\ldots ,0.95\right\}}\sum AP_{t}\left(c\right)$$

where t denotes the IoU threshold and C stands for the total number of categories.

FPS: The number of image frames the model processes in a second, or FPS, is a measure of inference speed, as defined in Equation 20. Forward propagation and post-processing (like NMS) are included in inference time, but data loading is not.

(20) $$\text{FPS}=\frac{1}{{\text{T}}_{\text{inference}}}$$

where the average inference time for each image is represented by T_inference_ (in seconds).

Parameters: The model’s total trainable parameter count was reported as the number of trainable parameters in the tables. For compact textual discussion, the same value was sometimes expressed in millions (M). Regarding convolutional layers, the parameter calculation is shown in Equation 21:

(21) $${\text{Params}}_{\text{conv}}=\text{Cout}\times \left({\text{C}}_{\text{in}}\times {\text{K}}^{2}+1\right)$$

where K is the size of the convolution kernel and +1 is the bias term, Cout and Cin stand for the number of output and input channels, respectively.

GFLOPs: Giga Floating Point Operations, or GFLOPs, are a measure of computational complexity that indicate how many floating-point operations the model has to perform in order to finish a single forward pass. For convolution layers, the corresponding computation is shown in Equation 22:

(22) $${\text{FLOPs}}_{\text{conv}}=2\times {\text{C}}_{\text{out}}\times {\text{C}}_{\text{in}}\times {\text{K}}^{2}\times \text{H}\times \text{W}$$

where the coefficient 2 denotes one multiplication and one addition operation, and H and W stand for the spatial dimensions of the output feature map.

Weight/MB: The model weight was reported as the storage size of the exported inference weight file in MB under the same export setting. This value was treated as an empirical storage-size metric rather than being calculated solely from the number of trainable parameters. The analytical estimate is shown in Equation 23.

(23) $$\text{weight}\left(\text{MB}\right)=\frac{\text{Parameters}\times {\text{Bits}}_{\text{per\_param}}}{8\times 1024^{2}}$$

where bits_per_param_ denotes the stored precision when an analytical estimate is used. In the tables, however, Weight/MB was measured directly from the exported inference weight files under the same export setting; therefore, it may not be exactly equal to a simple FP32 parameter-count estimate.

To preliminarily assess external-domain generalization, DFA-YOLO and YOLOv11-OBB were evaluated without fine-tuning on three public stomatal datasets: OilPalm/Kwong2021, LabelStoma/Casado2020, and the wheat leaf imprint stomatal dataset reported by Toda et al. (2021) (Casado-García et al., 2020; Bin et al., 2021; Toda et al., 2021). OilPalm/Kwong2021 and LabelStoma/Casado2020 each contributed 50 external test images, whereas the Toda et al. (2021) wheat dataset contained 728 microscopic images and 56, 494 annotated stomatal instances. None of these external images were used for training, hyperparameter tuning, threshold calibration, or model selection. Evaluation metrics included mAP50, mAP75, mAP95, mAP50–95, count MAE, count bias, and center-matching F1-score.

#### 2.5.1 Task-aligned comparison with StoManager1

To provide a task-aligned comparison with a specialized stomatal phenotyping system, StoManager1 was evaluated on the same original, non-augmented held-out multi-stomata test set as DFA-YOLO. This test set contained 50 maize stomatal microscopic images and 3, 959 manually annotated stomatal instances. The data split used for this comparison is provided in **Supplementary Table 2**. Because StoManager1 and DFA-YOLO differ in output format and target traits, OBB mAP was not used for this comparison. The comparison was therefore limited to shared count- and center-localization-related metrics, rather than the full morphometric measurement capacity of StoManager1. These metrics included stomatal count MAE, count RMSE, count bias, Pearson correlation coefficient, and center-matching precision, recall, and F1-score.

The confidence threshold of StoManager1 was selected using the validation set only. Thresholds around the validation optimum (0.001, 0.002, 0.003, 0.005, 0.010, 0.020, and 0.050) were evaluated, and conf = 0.005 was selected based on validation count MAE and center-matching F1-score. DFA-YOLO was first evaluated using the default inference setting recorded during the main detection experiments (conf = 0.12, IoU = 0.55, imgsz = 1024, max_det = 600). To provide a threshold-adjusted comparison for count-oriented phenotyping, a validation-set threshold sensitivity analysis was also performed for DFA-YOLO using confidence thresholds of 0.12, 0.15, 0.20, 0.25, 0.30, 0.35, 0.40, 0.45, 0.50, 0.60, 0.70, and 0.80. The threshold conf = 0.60 was selected based on validation count MAE and center-matching F1-score and then fixed for test-set evaluation. The test set was not used for threshold tuning.

For center-matching evaluation, ground-truth (GT) centers were derived from the manually annotated YOLO-OBB labels. A predicted stomatal center was considered a true positive if it fell inside a GT OBB or if the distance between the predicted center and the GT center was less than 0.5×min(w, h), where w and h denote the width and height of the corresponding GT OBB. One-to-one matching was enforced to avoid assigning multiple predictions to the same GT stomatal instance. Statistical significance for per-image absolute count errors was assessed using a paired two-sided Wilcoxon signed-rank test. Bootstrap resampling with 1, 000 iterations was used to estimate 95% confidence intervals for the improvements in count MAE and center-matching F1-score.

### 2.6 Experimental configuration

All experiments were conducted on an NVIDIA GeForce RTX 3090 GPU (24 GB) using PyTorch 2.0.0 and the Ultralytics YOLO implementation. Training was performed with an input size of 1024×1024 pixels and a batch size of 16. A two-stage training strategy was employed: the teacher model was trained for 100 epochs on the single-stomata dataset to learn clean stomatal features, followed by student model training for 150 epochs on the multi-stomata dataset with MGD distillation (λ=0.65). The AdamW optimizer was used with an initial learning rate of 0.001 and weight decay of 0.0005. Model evaluation used an IoU threshold of 0.5 and a default inference NMS IoU threshold of 0.55. Detailed experimental parameters are provided in **Supplementary Table 3**.

### 2.7 Case study: detection-oriented stomatal descriptor extraction under drought stress

A drought stress experiment was conducted using maize inbred line KN5585. After germination, seedlings were transferred to seedling trays and transplanted to individual pots at the two-leaf stage, with three plants per pot. For each genotype, three pots were assigned to the well-watered control (WW) group and three pots to the water-stressed (WS) group. Plants were cultivated in a greenhouse under controlled conditions. At the four-leaf stage, irrigation was withheld from the WS group while the WW group continued to receive regular watering. After 20 days of water withholding, when WS group leaves exhibited visible curling, leaf samples were collected from both groups. Stomatal imprints were obtained using the nail polish impression method as described in Section 2.2. A total of 90 images from the WW group and 70 images from the WS group were analyzed using DFA-YOLO. Statistical significance was assessed using independent samples t-tests (α = 0.05).

## 3 Results

### 3.1 Ablation studies

To systematically evaluate the contribution of each proposed component, ablation experiments were conducted on the multi-stomata test set. To isolate the effects of C3k2_AssemFormer and Focaler-CIoU from knowledge distillation, all models in **Table 2** were trained and evaluated without MGD or any other distillation method. Model 1 represents the YOLOv11-OBB baseline, Model 2 adds only C3k2_AssemFormer, Model 3 adds only Focaler-CIoU, and Model 4 combines C3k2_AssemFormer and Focaler-CIoU.

**Table 2:** Ablation study without knowledge distillation for isolating C3k2_AssemFormer and Focaler-CIoU.

Model	conv	Loss	P	R	mAP50	mAP75	mAP95	mAP50–95	FPS(img/s)	Parameters	GFLOPs	Weight/MB
1	×	×	0.923	0.786	0.872	0.775	0.074	0.671	69.7	2653918	6.6	6.2
2	✓	×	0.915	0.789	0.875	0.869	0.122	0.765	42.5	4054046	13.9	8.8
3	×	✓	0.925	0.873	0.910	0.866	0.104	0.786	69.2	2702206	6.8	6.3
4	✓	✓	0.921	0.874	0.915	0.869	0.115	0.813	42.0	4102334	14.1	8.9

P, Precision; R, Recall; mAP50, mAP75, and mAP95, mean Average Precision at IoU thresholds of 0.50, 0.75, and 0.95, respectively; mAP50–95, mean Average Precision averaged over IoU thresholds from 0.50 to 0.95; FPS, Frames Per Second; GFLOPs, Giga Floating Point Operations.

As shown in **Table 2**, C3k2_AssemFormer and Focaler-CIoU contributed independently without knowledge distillation. Compared with Model 1, adding C3k2_AssemFormer alone in Model 2 increased mAP75 from 0.775 to 0.869, mAP95 from 0.074 to 0.122, and mAP50–95 from 0.671 to 0.765, indicating improved high-overlap OBB localization. Adding Focaler-CIoU alone in Model 3 increased recall from 0.786 to 0.873, mAP50 from 0.872 to 0.910, and mAP50–95 from 0.671 to 0.786. The combined configuration, Model 4, achieved the highest recall (0.874), mAP50 (0.915), and mAP50–95 (0.813) among the no-distillation ablation settings. Model 2 achieved the highest mAP95 (0.122), whereas Model 3 achieved the highest precision (0.925) and maintained near-baseline FPS (69.2 vs. 69.7 in Model 1). These results indicate complementary effects of the two modules. The complexity increase was mainly introduced by C3k2_AssemFormer, which increased GFLOPs from 6.6 to 13.9, whereas Focaler-CIoU introduced only a small increase from 6.6 to 6.8 GFLOPs when used alone.

### 3.2 Comparative experiments

#### 3.2.1 Comparison of knowledge distillation strategies

To validate the effectiveness of knowledge distillation in cross-dataset transfer, a two-stage comparison was conducted. First, YOLOv11-OBB without distillation was compared with YOLOv11-OBB using MGD distillation. Subsequently, under the full model architecture with C3k2_AssemFormer and Focaler-CIoU as the student network, MGD was benchmarked against four alternative distillation methods (CWD, AT, PKD, and SKD), using the teacher network pre-trained on the single-stomata dataset. All experiments used identical configurations except for the distillation strategy.

**Figure 7:**
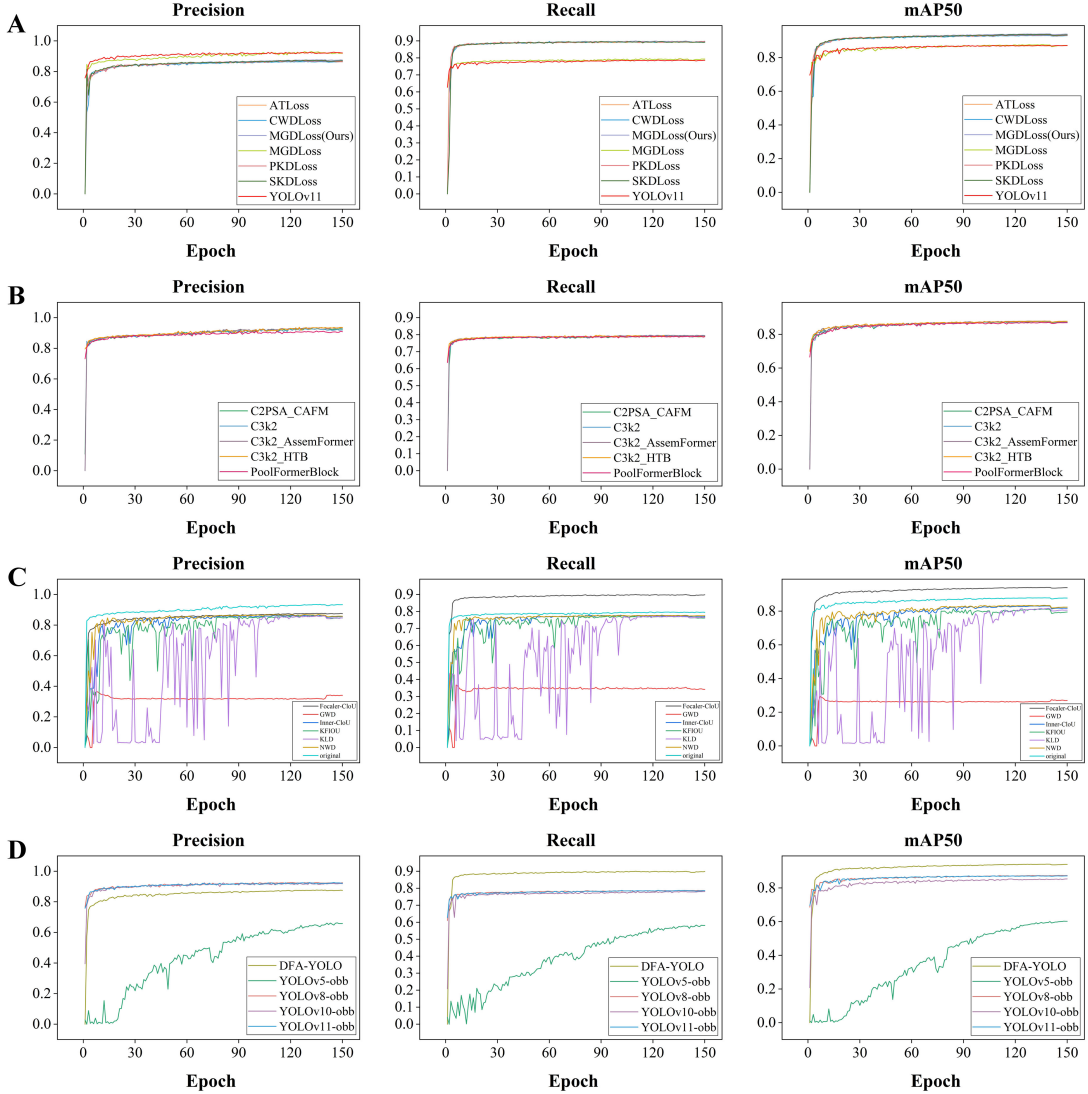
Comparative performance analysis of proposed modules and strategies. **(A)** Results of knowledge distillation comparison experiment. **(B)** Results of feature extraction module comparison experiment. **(C)** Results of loss function comparison experiment. **(D)** Results of the comparison with YOLO-series OBB detection baselines.

As shown in **Table 3** and **Figure 7A**, applying MGD to the YOLOv11-OBB baseline slightly improved recall, mAP50, mAP75, mAP95, and mAP50–95. In the full-model comparison, MGDLoss (Ours) achieved the highest recall (0.900), mAP50 (0.941), mAP75 (0.848), mAP95 (0.092), and mAP50–95 (0.741) among the tested distillation strategies. Because the differences between MGDLoss (Ours) and ATLoss were small for some metrics, paired bootstrap resampling was performed at the image level. The differences in recall and mAP50 were not statistically significant (p = 0.108 and p = 0.350), whereas significant improvements were observed for mAP75, mAP95, and mAP50–95 (p = 0.028, p < 0.001, and p < 0.001, respectively; **Supplementary Table 4**). Compared with the no-distillation combined configuration in **Table 2**, MGD improved recall and mAP50 but reduced mAP75, mAP95, and mAP50–95. These results suggest that MGD mainly enhanced dense-scene coverage, while its advantage over ATLoss was supported primarily by stricter localization metrics rather than by mAP50 alone.

**Table 3:** Comparison of knowledge distillation methods.

Model core	P	R	mAP50	mAP75	mAP95	mAP50–95	FPS(img/s)	Parameters	GFLOPs	Weight/MB
None(YOLOv11)	0.923	0.786	0.872	0.775	0.074	0.671	69.7	2653918	6.6	6.2
MGDLoss	0.929	0.795	0.878	0.779	0.077	0.673	100.2	2653918	6.6	6.2
CWDLoss	0.864	0.894	0.931	0.731	0.060	0.630	56.6	4102334	14.1	8.9
ATLoss	0.876	0.892	0.940	0.768	0.064	0.662	61.3	4102334	14.1	8.9
PKDLoss	0.868	0.896	0.934	0.736	0.059	0.634	69.0	4102334	14.1	8.9
SKDLoss	0.873	0.893	0.937	0.783	0.072	0.678	52.6	4102334	14.1	8.9
MGDLoss(Ours)	0.875	0.900	0.941	0.848	0.092	0.741	44.9	4102334	14.1	8.9

P, Precision; R, Recall; mAP50, mAP75, and mAP95, mean Average Precision at IoU thresholds of 0.50, 0.75, and 0.95, respectively; mAP50–95, mean Average Precision averaged over IoU thresholds from 0.50 to 0.95; FPS, Frames Per Second; GFLOPs, Giga Floating Point Operations.

#### 3.2.2 Comparative study of feature extraction modules

To evaluate the effectiveness of C3k2_AssemFormer, it was compared with the original C3k2 module and several alternative feature extraction modules, including C2PSA_CAFM, C3k2_HTB, and PoolFormerBlock. All experiments maintained identical training configurations except for the feature extraction module.

As shown in **Table 4** and **Figure 7B**, C3k2_AssemFormer achieved the highest mAP50 (0.880), mAP75 (0.793), and mAP50–95 (0.686) among the evaluated feature extraction modules. Compared with the original C3k2 module, it improved precision from 0.929 to 0.935, mAP75 from 0.779 to 0.793, and mAP50–95 from 0.673 to 0.686, while recall remained unchanged at 0.795. However, its mAP95 was slightly lower than that of C3k2 (0.076 vs. 0.077), and the computational cost increased from 6.6 to 13.9 GFLOPs. These results indicate that C3k2_AssemFormer improved most detection metrics, but with higher computational cost and no improvement at the strictest IoU threshold.

**Table 4:** Results of comparative metrics for feature extraction modules.

Conv	P	R	mAP50	mAP75	mAP95	mAP50–95	FPS(img/s)	Parameters	GFLOPs	Weight/MB
C3k2	0.929	0.795	0.878	0.779	0.077	0.673	100.2	2653918	6.6	6.2
C2PSA_CAFM	0.929	0.792	0.876	0.773	0.071	0.661	94.7	2711199	6.6	6.0
C3k2_HTB	0.937	0.790	0.879	0.780	0.068	0.672	67.8	4031786	14.3	8.7
PoolFormerBlock	0.907	0.790	0.870	0.770	0.069	0.667	77.1	2695747	6.9	5.9
C3k2_AssemFormer	0.935	0.795	0.880	0.793	0.076	0.686	72.1	4054046	13.9	8.8

P, Precision; R, Recall; mAP50, mAP75, and mAP95, mean Average Precision at IoU thresholds of 0.50, 0.75, and 0.95, respectively; mAP50–95, mean Average Precision averaged over IoU thresholds from 0.50 to 0.95; FPS, Frames Per Second; GFLOPs, Giga Floating Point Operations.

#### 3.2.3 Experiment for comparing loss functions

To evaluate the effect of Focaler-CIoU, it was compared against several oriented bounding box loss-related regression formulations, including Inner-CIoU, NWD, KLD, KFIoU, and GWD. All experiments used identical training configurations except for the loss-related regression module. **Table 5** presents the experimental findings.

**Table 5:** Comparison of loss function.

Loss	P	R	mAP50	mAP75	mAP95	mAP50–95	FPS(img/s)	Parameters	GFLOPs	Weight/MB
original	0.935	0.795	0.880	0.793	0.076	0.686	72.1	4054046	13.9	8.8
Inner-CIoU	0.863	0.775	0.831	0.502	0.034	0.429	63.2	4102334	14.1	8.9
NWD	0.868	0.777	0.835	0.597	0.042	0.512	57.9	4102334	14.1	8.9
KLD	0.858	0.770	0.818	0.338	0.023	0.289	58.8	4102334	14.1	8.9
KFIoU	0.861	0.769	0.813	0.326	0.022	0.275	58.5	4102334	14.1	8.9
GWD	0.355	0.342	0.284	0.062	0.000	0.074	58.6	4102334	14.1	8.9
Focaler-CIoU(Ours)	0.875	0.900	0.941	0.848	0.092	0.741	44.9	4102334	14.1	8.9

P, Precision; R, Recall; mAP50, mAP75, and mAP95, mean Average Precision at IoU thresholds of 0.50, 0.75, and 0.95, respectively; mAP50–95, mean Average Precision averaged over IoU thresholds from 0.50 to 0.95; FPS, Frames Per Second; GFLOPs, Giga Floating Point Operations.

As shown in **Table 5** and **Figures 7C**, Focaler-CIoU achieved the highest recall (0.900), mAP50 (0.941), mAP75 (0.848), mAP95 (0.092), and mAP50–95 (0.741) among the compared loss functions. Compared with the original loss setting, it improved recall by 10.5 percentage points and mAP50–95 by 5.5 percentage points, while precision decreased from 0.935 to 0.875. Thus, Focaler-CIoU improved stomatal coverage and localization across multiple IoU thresholds, but introduced more false-positive predictions.

To further assess whether Focaler-CIoU depended on a single upper-threshold setting, an additional controlled sensitivity analysis was conducted for u_stomata_. To isolate the threshold effect, this analysis was performed under the YOLOv11-OBB + Focaler-CIoU setting, without MGD distillation or other additional DFA-YOLO components. As shown in **Supplementary Table 5**, mAP50–95 remained stable across u_stomata_=0.900–0.975, varying only from 0.781 to 0.786. The setting u_stomata_=0.95 was within the best-performing range rather than an isolated optimum, indicating that the observed effect was not dependent on a single selected threshold value.

The GWD loss showed a marked performance drop in this experiment, with mAP50, mAP75, and mAP50–95 decreasing to 0.284, 0.062, and 0.074, respectively. This may be related to the dense stomatal imaging scenario, where stomata are small, elongated, closely adjacent, and often have blurred boundaries. Under this setting, the Gaussian-distribution-based box regression may provide insufficient discrimination among neighboring stomata, leading to degraded localization performance.

#### 3.2.4 Comparison with YOLO-series OBB detection baselines

To evaluate DFA-YOLO under a consistent OBB detection setting, we compared it with four YOLO-series OBB baselines, including YOLOv5-OBB, YOLOv8-OBB, YOLOv10-OBB, and YOLOv11-OBB. All models were trained and evaluated on the same maize stomatal dataset using identical data splits, input resolution, and experimental settings. This comparison was designed to assess OBB-based object detection performance rather than to claim superiority over specialized stomatal phenotyping systems such as StomataCounter, DeepStomata, StomaAI, StoManager1, or LeafNet, which differ in task objectives, annotation formats, output types, and morphometric capabilities.

As shown in **Table 6** and **Figures 7D**, DFA-YOLO achieved the highest recall (0.900), mAP50 (0.941), mAP75 (0.848), and mAP50–95 (0.741) among the YOLO-series OBB baselines. Compared with YOLOv11-OBB, DFA-YOLO improved recall, mAP50, mAP75, mAP95, and mAP50–95, but precision decreased from 0.923 to 0.875. YOLOv10-OBB achieved a slightly higher mAP95 than DFA-YOLO (0.098 vs. 0.092). These results indicate that DFA-YOLO improved dense stomatal detection and OBB localization across most IoU thresholds. Because these metrics evaluate rotated-box overlap, they reflect OBB-level localization performance rather than pixel-level shape accuracy or segmentation-level morphometric performance. This improvement was accompanied by a measurable precision trade-off: on the held-out maize multi-stomata test set, false positives increased from 5.19 to 10.18 per image, and count bias shifted from −11.75 to +2.26 stomata per image, with a corresponding increase in density overestimation (**Supplementary Table 9**).

**Table 6:** Comparison with YOLO-series OBB detection baselines under identical experimental settings.

Model	P	R	mAP50	mAP75	mAP95	mAP50–95	FPS(img/s)	Parameters	GFLOPs	Weight/MB
YOLOv5-OBB	0.556	0.516	0.530	0.364	0.002	0.366	143.3	2004058	4.9	4.7
YOLOv8-OBB	0.925	0.786	0.874	0.841	0.097	0.730	134.5	3082710	8.4	6.6
YOLOv10-OBB	0.920	0.780	0.853	0.843	0.098	0.735	102.9	2658214	8.0	7.2
YOLOv11-OBB	0.923	0.786	0.872	0.775	0.074	0.671	69.7	2653918	6.6	6.2
DFA-YOLO	0.875	0.900	0.941	0.848	0.092	0.741	44.9	4102334	14.1	8.9

P, Precision; R, Recall; mAP50, mAP75, and mAP95, mean Average Precision at IoU thresholds of 0.50, 0.75, and 0.95, respectively; mAP50–95, mean Average Precision averaged over IoU thresholds from 0.50 to 0.95; FPS, Frames Per Second; GFLOPs, Giga Floating Point Operations.

Therefore, the practical value of DFA-YOLO lies in improving dense-stomata coverage and count-oriented localization within an OBB-based phenotyping workflow, rather than in providing the fastest inference speed or segmentation-level morphometric precision.

This comparison was limited to YOLO-series OBB detectors. Specialized stomatal phenotyping systems such as StomataCounter, DeepStomata, StomaAI, StoManager1, and LeafNet target different outputs, including counting, segmentation, pore measurement, and detailed morphometric extraction. A task-aligned comparison with StoManager1 is provided in Section 3.2.5.

In addition to the in-domain YOLO-series comparison, we conducted zero-shot external validation on three public stomatal datasets to examine whether model performance was restricted to the original maize imaging domain. As shown in **Supplementary Table 6**, DFA-YOLO achieved higher mAP50, mAP50–95, and Center F1 than YOLOv11-OBB on all three datasets. On OilPalm/Kwong2021, DFA-YOLO improved mAP50 from 0.507 to 0.633, mAP50–95 from 0.324 to 0.403, and Center F1 from 0.064 to 0.494; however, it still showed a negative count bias of -22.26 stomata per image, suggesting systematic undercounting under cross-domain differences in stomatal morphology and image appearance relative to the maize training domain. On LabelStoma/Casado2020, it improved mAP50 from 0.459 to 0.505 and Center F1 from 0.276 to 0.409. On the Toda et al. (2021) wheat leaf imprint stomatal dataset, DFA-YOLO further improved mAP50 from 0.532 to 0.550, mAP75 from 0.344 to 0.496, mAP50–95 from 0.326 to 0.394, reduced count MAE from 69.55 to 51.93, reduced count bias from -69.53 to -46.15 stomata per image, and increased Center F1 from 0.180 to 0.451. These results provide stronger preliminary evidence of external-domain transferability beyond the internal maize dataset, although broad cross-domain generalization remains to be further validated.

Five-fold plant-wise cross-validation was further conducted to assess robustness beyond a single 7:2:1 split. As shown in **Supplementary Table 7**, DFA-YOLO increased mean mAP50–95 from 0.683 ± 0.080 to 0.701 ± 0.094 relative to YOLOv11-OBB. However, the fold-wise paired Wilcoxon signed-rank test was not statistically significant (p = 0.188), indicating a numerically favorable but statistically inconclusive improvement.

#### 3.2.5 Task-aligned comparison with StoManager1

A task-aligned comparison was conducted between DFA-YOLO and StoManager1 on the original, non-augmented held-out multi-stomata test set, which contained 50 maize stomatal microscopic images and 3, 959 manually annotated stomatal instances. Because the two methods produce different outputs, OBB mAP was not used; instead, stomatal count consistency and center-matching localization performance were evaluated.

As shown in **Supplementary Table 8**, validation-calibrated DFA-YOLO showed closer agreement with manual annotations than StoManager1 for the shared count- and center-matching metrics. It reduced count MAE from 28.66 to 8.22, RMSE from 38.06 to 10.06, and count bias from -17.06 to 4.50 stomata per image, while increasing Pearson correlation from 0.509 to 0.977. For center matching, DFA-YOLO improved precision, recall, and F1-score from 0.713, 0.560, and 0.627 to 0.892, 0.943, and 0.917, respectively. The paired two-sided Wilcoxon signed-rank test showed a significant reduction in per-image absolute count error (p=2.12×10^−9^). Bootstrap analysis with 1, 000 resamplings showed an MAE improvement of 20.44 (95% CI: 14.00–27.38) and a center-matching F1-score improvement of 0.290 (95% CI: 0.242–0.354).

These results demonstrate the applicability of DFA-YOLO to dense maize stomatal localization and count estimation. As DFA-YOLO outputs oriented bounding boxes, this comparison focused on count consistency and center-level localization, while pore contours, guard-cell boundaries, and fine-scale morphometric traits remained beyond the scope of this evaluation.

#### 3.2.6 Manual annotation validation

**Figure 8:**
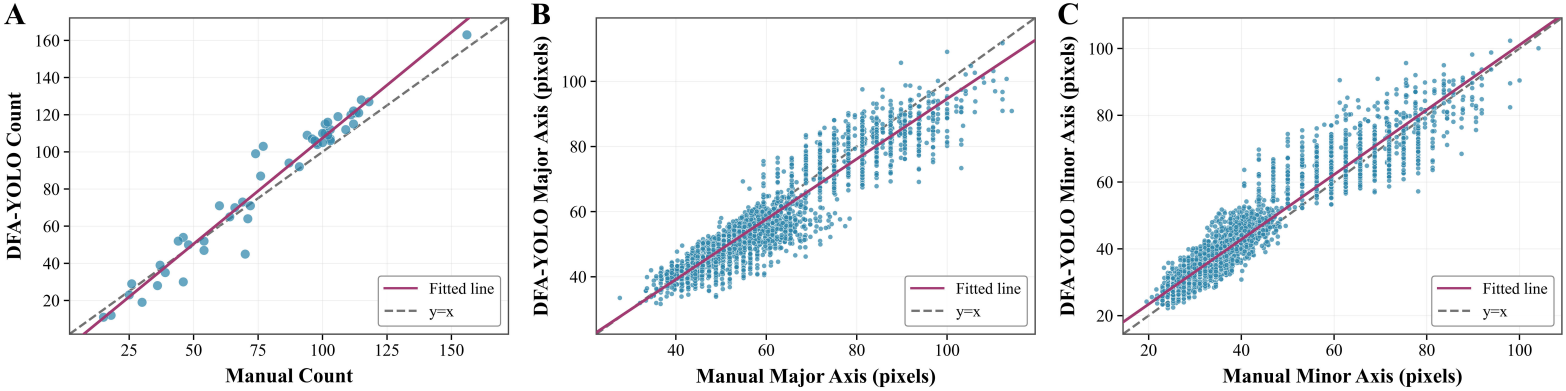
Agreement between manual OBB annotations and DFA-YOLO predictions. **(A)** Stomatal count (per-image basis, n = 50). **(B)** OBB-derived major axis length (per-stoma basis, n = 3, 730 matched pairs, IoU ≥ 0.5). **(C)** OBB-derived minor axis length (per-stoma basis, n = 3, 730 matched pairs, IoU ≥ 0.5). Purple-red lines, fitted regression; dark gray dashed lines, identity line (y = x).

Model predictions were validated against manual OBB annotations on the complete held-out multi-stomata test set, including 50 original images and 3, 959 manually annotated stomata. As shown in **Figure 8A**, using the validation-calibrated inference setting, DFA-YOLO showed strong count agreement with manual annotations (Pearson r = 0.977, p < 0.001; RMSE = 10.06; MAE = 8.22; bias = 4.50 stomata per image). For OBB-derived dimensional descriptors, 3, 730 stomata (94.2%) were successfully matched using IoU ≥ 0.5. Major and minor axis lengths also showed strong agreement with manual OBB annotations (**Figures 8B, C**), with Pearson r values of 0.945 and 0.953 and average biases of −2.28 and 2.75 pixels, respectively. Detailed RMSE, MAE, bias, Bland–Altman 95% limits of agreement, correlation statistics, and matched-pair counts are provided in **Supplementary Table 10**.

#### 3.2.7 Case study: detection-oriented stomatal descriptor extraction under drought stress

To evaluate the practical use of DFA-YOLO for downstream detection-oriented descriptor extraction, maize (KN5585) leaves from two water treatments were analyzed: well-watered control (WW, n=90 images) and water-stressed (WS, n=70 images). The analysis focused on stomatal density and coarse OBB-derived geometric descriptors, including stomatal area, major axis length, and minor axis length.

**Figure 9:**
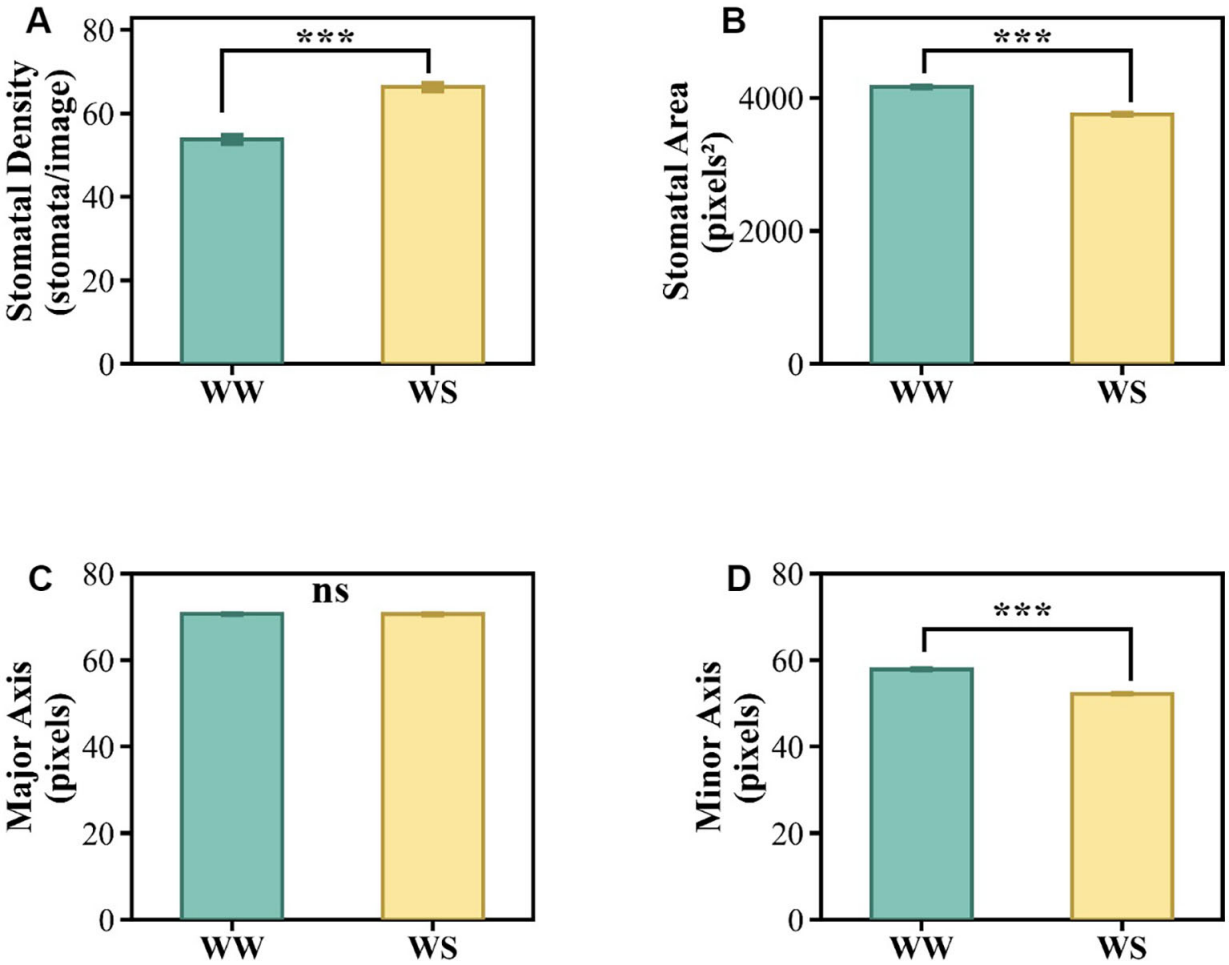
Comparison of detection-oriented stomatal descriptors between well-watered and drought stress conditions. **(A)** Stomatal density. **(B)** OBB-derived stomatal area. **(C)** OBB-derived major axis length. **(D)** OBB-derived minor axis length. Bars represent mean values, and error bars represent standard error of the mean (SEM). Asterisks indicate statistical significance (***P < 0.001; ns, not significant). WW, well-watered control (n=90 images); WS, water stress (n=70 images).

As shown in **Supplementary Table 11** and **Figure 9**, the WS group showed a 23.4% higher stomatal density than the WW group (from 53.74 to 66.30 stomata/image, p < 0.001). In contrast, OBB-derived stomatal area was 9.9% lower in the WS group (from 4163.39 to 3751.37 pixels², p < 0.001). This change was accompanied by a decrease in OBB-derived minor axis length (from 57.87 to 52.21 pixels, 9.8% reduction, p < 0.001), whereas OBB-derived major axis length remained similar between treatments (70.67 vs. 70.64 pixels, p = 0.889). The direction of these changes was consistent with expected stomatal responses to water stress and demonstrates that DFA-YOLO can support rapid extraction of treatment-associated detection-oriented descriptors.

## 4 Discussion

### 4.1 Practical effectiveness of domain-specific adaptations

The ablation results indicate that DFA-YOLO benefited from the combined use of task-specific adaptations for dense maize stomatal detection. Cross-dataset distillation, C3k2_AssemFormer, and Focaler-CIoU addressed complementary aspects of the task: structural guidance from isolated stomata, contextual representation for dense arrangements, and gradient reweighting for difficult positive samples. These adaptations improved dense-scene detection and OBB localization while building on established techniques, including knowledge distillation, IoU-based loss reweighting, CNN feature extraction, and linear attention.

Together, these findings indicate that the performance gains of DFA-YOLO mainly arise from task-specific integration of established techniques for dense stomatal localization. The gains in recall and mAP were accompanied by lower precision and higher computational cost, indicating an accuracy–efficiency trade-off under the tested maize imaging workflow.

### 4.2 Overcoming limitations of current approaches

The YOLO-series baseline comparison provides the main evidence for interpreting the limitations of general-purpose OBB detectors in this task. As shown in **Table 6**, YOLOv11-OBB achieved 0.786 recall and 0.872 mAP50, whereas DFA-YOLO achieved 0.900 recall and 0.941 mAP50 under the same maize stomatal dataset and evaluation setting. These results suggest that dense maize stomatal images require stronger coverage of small and densely distributed objects than the baseline detector provided. Because the comparison was conducted within one maize imaging workflow, this finding is task-specific and does not support a general conclusion about all YOLO-based OBB detectors.

Previous bounding-box-based stomatal detectors provide useful context for interpreting DFA-YOLO, although their reported results are not directly comparable because of differences in datasets, annotation formats, imaging conditions, and evaluation protocols. LabelStoma reported 85.3% mAP50 on relatively simple stomatal images (Casado-García et al., 2020), and I3-YOLOv8s reported 89.2% mAP50 for irregularly shaped stomata (Gong et al., 2025). In comparison, DFA-YOLO achieved 94.1% mAP50 on the present dense maize multi-stomata dataset. These results suggest that the proposed OBB-based adaptations were effective under the tested maize imaging workflow, while the cross-study comparison remains descriptive because of differences in datasets, annotation formats, imaging conditions, and evaluation protocols.

These comparisons are most appropriately viewed within the scope of bounding-box-based stomatal detection. OBB-based detection and segmentation-based or hybrid stomatal phenotyping differ in output format and morphometric scope. Systems such as StoManager1 and LeafNet address broader morphometric objectives than DFA-YOLO. StoManager1 estimates multiple stomatal and guard-cell traits using CNN-based detection combined with empirical and geometrical algorithms, whereas LeafNet supports stomatal localization, pavement-cell segmentation, and extraction of epidermal morphological parameters. These systems are more appropriate when pore contours, aperture area, guard-cell morphology, pavement-cell geometry, or instance-level shape information are required. In contrast, DFA-YOLO predicts rotated rectangular regions that approximate stomatal position, size, and orientation. Therefore, DFA-YOLO is more appropriate for efficient stomatal localization, density estimation, and preliminary orientation-aware trait extraction, whereas segmentation-based workflows remain necessary for detailed morphometric analysis.

The comparison with StoManager1 further highlights the advantage of DFA-YOLO in detection-oriented stomatal phenotyping. On the original, non-augmented multi-stomata test set, validation-calibrated DFA-YOLO achieved lower count error, higher count correlation, and higher center-matching F1-score under the shared count- and center-localization metrics. This demonstrates its effectiveness for high-throughput localization and count estimation in dense maize stomatal images. The two workflows have different task objectives: DFA-YOLO focuses on efficient OBB-based detection and preliminary trait extraction, whereas complete segmentation-level benchmarking requires pixel-level masks, segmentation-oriented metrics, and direct evaluation of pore contours, aperture area, and guard-cell morphology. Therefore, segmentation- or measurement-oriented tools such as StoManager1 remain important for fine-scale pore and guard-cell morphometry.

The higher-threshold metrics further clarify the scope of DFA-YOLO. Although DFA-YOLO improved mAP75 and mAP50–95 compared with YOLOv11-OBB, these metrics still measure OBB overlap rather than pixel-level shape agreement. Thus, these metrics characterize OBB-level localization rather than segmentation-level morphometric accuracy. Because DFA-YOLO predicts oriented bounding boxes rather than masks, pore contours, aperture area, guard-cell boundary morphology, and instance-level shape accuracy were not evaluated in this study.

The component-level experiments provide a direct basis for interpreting the contribution of each adaptation. In the no-distillation ablation study (**Table 2**), C3k2_AssemFormer mainly improved stricter OBB localization metrics, including mAP75, mAP95, and mAP50–95, whereas Focaler-CIoU mainly improved recall, mAP50, and mAP50–95. Their combination achieved the highest recall, mAP50, and mAP50–95 among the no-distillation ablation settings, but not the highest mAP95 or FPS. The separate comparisons in **Tables 3**-**5** further supported complementary roles of the three adaptations: MGD improved recall-oriented dense-scene coverage, C3k2_AssemFormer improved feature aggregation, and Focaler-CIoU improved loss-related regression performance. Overall, the observed gains appear to result from complementary contributions of distillation, feature aggregation, and loss reweighting, with measurable accuracy–efficiency and coverage–localization trade-offs.

### 4.3 Accuracy–efficiency trade-off

DFA-YOLO achieved 94.1% mAP50, 84.8% mAP75, 74.1% mAP50–95, and 90.0% recall with 4.10M parameters, but this improvement was accompanied by lower FPS and higher computational cost. Together, these results indicate an accuracy–efficiency trade-off, with improved recall and mAP accompanied by lower FPS and higher computational cost.

In high-throughput stomatal phenotyping, missed detections can affect downstream count- and density-based analyses. Because stomatal traits are closely associated with photosynthetic performance, water-use efficiency, and stress responses (Carminati et al., 2020; Cernusak and De Kauwe, 2021; Buckley, 2023), improved recall and mAP50 support detection-oriented trait extraction, although this comes with increased computational cost.

The no-distillation ablation study (**Table 2**) further clarifies the trade-off. C3k2_AssemFormer improved mAP75, mAP95, and mAP50–95 but reduced FPS because GFLOPs increased from 6.6 to 13.9. Focaler-CIoU improved recall, mAP50, and mAP50–95 while introducing only a small complexity increase relative to the baseline, with parameters increasing from 2.65M to 2.70M and GFLOPs increasing from 6.6 to 6.8. Their combination achieved the highest recall, mAP50, and mAP50–95 among the no-distillation ablation settings, but not the highest mAP95 or FPS.

After adding MGD, the final DFA-YOLO further improved recall and mAP50, supporting dense-scene coverage and count-oriented localization, although stricter localization metrics were lower than those of the no-distillation Model 4 configuration. Compared with YOLO-series OBB baselines (**Table 6**), DFA-YOLO achieved higher recall, mAP50, mAP75, and mAP50–95, but with lower FPS and higher computational cost. Therefore, its practical value lies primarily in improving dense-stomata coverage and count-oriented localization rather than providing the fastest inference speed.

### 4.4 System implementation and agronomic application

Stomatal density and size-related traits are closely associated with crop photosynthetic efficiency and water-use efficiency (Carminati et al., 2020; Cernusak and De Kauwe, 2021; Buckley, 2023). Traditional manual counting severely limits large-scale germplasm screening due to its time-consuming, labor-intensive, and highly subjective nature (Ghannoum et al., 2025; Jin et al., 2025). In this context, DFA-YOLO reduces single-image processing time to 0.02 seconds and provides orientation-aware bounding boxes for automated stomatal localization, density estimation, and coarse size- and orientation-related trait extraction. These OBB-derived descriptors can support preliminary high-throughput screening of maize stomatal traits, particularly for count-, density-, size-, and orientation-related comparisons. More detailed morphometric traits, including pore contour, aperture geometry, and guard-cell boundary morphology, belong to segmentation-level phenotyping and require segmentation-based validation or hybrid detection–segmentation analysis.

Manual annotation validation further supported this detection-oriented use case. In the multi-stomata test set, DFA-YOLO showed agreement with manual OBB annotations for stomatal counting and OBB-derived dimensional descriptors, supporting count-oriented analysis and coarse geometric description under the tested maize imaging workflow.

The drought-stress case study illustrates a downstream use of these descriptors. The WS group showed higher stomatal density and lower OBB-derived stomatal area and minor axis length than the WW group, while OBB-derived major axis length remained similar between treatments. These expected treatment-associated patterns support the use of DFA-YOLO for screening-oriented comparison of stomatal count and coarse OBB-derived geometric descriptors under controlled imaging conditions.

These results suggest that DFA-YOLO can serve as a detection-oriented tool for high-throughput maize stomatal localization and preliminary trait extraction under the tested imaging conditions. This study provides an OBB-based framework for dense stomatal localization and preliminary trait extraction under practical maize microscopic imaging conditions. To support practical use, we developed an intelligent stomatal detection and analysis platform that integrates model inference, visualization, and data export. The platform supports YOLO-series models, multi-format output, and both single-image and batch detection modes. Further validation across additional imaging workflows will be needed before broader deployment.

Although this work improves OBB-based maize stomatal detection under the tested imaging conditions, several limitations remain. The number of original microscopic images remains limited for deep learning. Although training-set augmentation, cross-dataset distillation, and regularization were used to reduce overfitting risk, augmented images were not treated as independent biological samples. First, the present framework is formulated as an OBB-based detection task. This design supports efficient stomatal localization, density estimation, orientation description, and preliminary OBB-derived trait extraction, but it does not provide segmentation-level morphometry. Accordingly, full segmentation-level benchmarking against tools such as LeafNet, segmentation-oriented outputs of StoManager1, and modern lightweight segmentation models remains a future extension requiring segmentation-oriented annotations and evaluation metrics. Precise analysis of pore geometry, aperture traits, guard-cell boundaries, or pavement-cell morphology will require segmentation-based validation or hybrid detection–segmentation workflows in future studies. Second, external generalizability remains limited. Zero-shot evaluation on OilPalm/Kwong2021, LabelStoma/Casado2020, and the wheat leaf imprint stomatal dataset of Toda et al. (2021) showed that DFA-YOLO consistently achieved higher mAP50, mAP50–95, and Center F1 than YOLOv11-OBB, and on the larger wheat dataset also obtained higher mAP75 together with lower count MAE and smaller count bias. Nevertheless, the remaining count errors, the low absolute mAP95 values, and the heterogeneity of species, microscopes, illumination conditions, and epidermal impression protocols indicate that broad cross-domain generalization has not yet been established. A representative example is OilPalm/Kwong2021, on which DFA-YOLO still showed a count bias of -22.26 stomata per image. We hypothesize that this undercount mainly arose from systematic false negatives under domain shift: relative to the maize training domain, differences in species-specific stomatal morphology, apparent scale, inter-stomatal spacing, contrast, and imaging conditions likely reduced confidence for some true stomata and caused missed detections. This suggests that count accuracy may remain more sensitive than detector-level overlap metrics under cross-domain transfer. In addition, StomataCounter, DeepStomata, and LeafNet were not included in quantitative comparison because their outputs, required annotations, and evaluated traits are not directly aligned with the present OBB-based detection setting: StomataCounter is primarily counting-oriented, whereas DeepStomata and LeafNet rely on segmentation-level outputs for aperture or epidermal morphometric analysis. By contrast, StoManager1 allowed a limited task-aligned comparison through shared count- and center-localization metrics. A quantitatively fair benchmark against the excluded systems would require task-aligned annotations and metrics, such as pixel-level masks and segmentation-oriented morphometric evaluation, which were outside the scope of the present study. Further validation is still needed across additional species and taxonomic groups, including rice, soybean, forest tree species, and both monocots and dicots, as well as across microscopes, magnifications, imaging modalities, public datasets, and tissue types. Third, performance optimization remains feasible in extreme scenarios. The O(N) complexity of linear attention mitigates efficiency concerns compared to standard O(N²) attention; however, additional validation is needed for scalability on higher-resolution images or extremely dense stomatal scenes.

Future work will prioritize broader external validation using multi-species and multi-source stomatal datasets collected under diverse imaging and sample-preparation conditions. Based on these expanded evaluations, domain adaptation methods will be explored to improve cross-domain transferability (Jin et al., 2025). We will also explore multi-modal fusion and physiological modeling based on detection-oriented stomatal traits (Amsbury et al., 2016; Asif et al., 2025; Cheng et al., 2025).

## 5 Conclusion

This study developed and evaluated DFA-YOLO, an enhanced YOLOv11-OBB model for dense maize stomatal localization under the tested laboratory imaging workflow. DFA-YOLO achieved 94.1% mAP50, 84.8% mAP75, 74.1% mAP50–95, and 90.0% recall on the present maize dataset, and showed better preliminary external-domain performance than YOLOv11-OBB on public stomatal datasets. These results indicate improved OBB-based detection performance and preliminary external-domain transferability, while broad cross-domain generalization remains to be further validated. The improved detection performance was accompanied by increased computational cost; therefore, the full DFA-YOLO model is more suitable when recall and dense-scene coverage are prioritized, whereas lightweight alternative configurations may be more appropriate for speed-sensitive deployment. To support practical use, we deployed a prototype platform offering flexible inference modes, including the full DFA-YOLO model and lightweight alternatives.

## Data Availability

The raw data supporting the conclusions of this article will be made available by the authors, without undue reservation.
