# Correction: DFA-YOLO: an enhanced YOLOv11-OBB and knowledge distillation-based maize stomata detection system

**DOI:** 10.3389/fpls.2026.1921044

**Published:** 2026-07-06

**Authors:** Zhenzhen Lin, Rui Liu, Antong Deng, Zhongxiang Xing, Huajuan Gao, Ziqi Yang, Yanxi Liu, Yuling Liu, Zhuolun He, Binhong Zhou, Jie Xu, Yan Guo, Zhiyong Li

**Affiliations:** 1 College of Information Engineering, Sichuan Agricultural University, Ya’an, Sichuan, China; 2 College of Mechanical and Electrical Engineering, Sichuan Agricultural University, Ya’an, Sichuan, China; 3 Maize Research Institute, Sichuan Agricultural University, Wenjiang, Sichuan, China; 4 State Key Laboratory of Crop Gene Exploration and Utilization in Southwest China, Sichuan Agricultural University, Wenjiang, Sichuan, China

**Keywords:** detection platform, focaler-CIoU, knowledge distillation, stomatal detection, YOLOv11-OBB

The affiliation “College of Mechanical and Electrical Engineering, Sichuan Agricultural University, Ya’ an, Sichuan, China” was omitted for author “Zhuolun He”. This affiliation has now been added as affiliation 2 and the subsequent affiliations re-numbered.

“Zhuolun He” was erroneously assigned to affiliation “College of Information Engineering, Sichuan Agricultural University, Ya’an, Sichuan, China”. This affiliation has now been removed for author “Zhuolun He”.

The original version of this article has been updated.

