# Supplementary material for: DFA-YOLO: an enhanced YOLOv11-OBB and knowledge distillation-based maize stomata detection system
Source: Front Plant Sci. 2026 Jun 26;17:1852592. doi: 10.3389/fpls.2026.1852592 (PMC13382967; doi:10.3389/fpls.2026.1852592)
Supplement: Supplementary file 1 [file DataSheet1.pdf]

Supplementary Material

Table S1. Dataset statistics.

Index	Single stoma	Multiple stoma	Aggregate
Original image	551	502	1053
Enhancement method	9 types	6 types	-
Enhanced image	1491	2106	3597
Expansion ratio	2.70×	4.20×	3.42×

Table S2. Plant-wise dataset split used for the task-aligned comparison with StoManager1. The original 502 multi-stomata images were grouped by plant identity and then assigned to training, validation, and test sets at an approximate ratio of 7:2:1. All images from the same plant were kept within the same subset. Data augmentation was applied only to the plant-wise training set, whereas validation and test images remained original and non-augmented. The validation set was used for threshold calibration, and the test set was used for the final task-aligned comparison.

Split	Original images	Augmented images	Final images
Training	352	1604	1956
Validation	100	0	100
Test	50	0	50
Total	502	1604	2106

Table S3. Parameters of the experimental configuration.

Configuration	Parameter
Hardware platform	NVIDIA GeForce RTX 3090 (24GB)
Framework	PyTorch 2.0.0
YOLO implementation	Ultralytics YOLO implementation
Input size	1024x1024
Batch size	16
Optimizer	AdamW
Initial learning rate	0.001
Weight decay	0.0005
Teacher training epochs	100
Student training epochs	150
MGD mask ratio	$\lambda = 0.65$
IoU threshold	0.5
Default inference NMS IoU threshold	0.55

Table S4. Statistical comparison between ATLoss and MGDLoss(Ours) using image-level paired bootstrap resampling. R: Recall; mAP50, mAP75, and mAP95: mean Average Precision at IoU thresholds of 0.50, 0.75, and 0.95, respectively; mAP50 – 95: mean Average Precision averaged over IoU thresholds from 0.50 to 0.95; CI: confidence interval. Difference was calculated as MGDLoss(Ours) − ATLoss. The 95% bootstrap CI and P-value were estimated from 1,000 paired bootstrap resamples of the held-out multi-stomata test images.

Metric	Difference (MGDLoss(Ours) − ATLoss)	95% bootstrap CI	P-value
R	0.008	[-0.001, 0.015]	0.108
mAP50	0.001	[-0.001, 0.003]	0.350
mAP75	0.080	[0.005, 0.152]	0.028
mAP95	0.028	[0.010, 0.045]	<0.001
mAP50–95	0.079	[0.034, 0.120]	<0.001

Table S5. Sensitivity analysis of the fixed upper threshold $u_{stomata}$ in Focaler-CIoU. The lower threshold was fixed at $d_{stomata}$=0.00. P: Precision; R: Recall; mAP50, mAP75, and mAP95: mean Average Precision at IoU thresholds of 0.50, 0.75, and 0.95, respectively; mAP50–95: mean Average Precision averaged over IoU thresholds from 0.50 to 0.95.

$u_{stomata}$	P	R	mAP50	mAP75	mAP95	mAP50-95
0.900	0.915	0.884	0.912	0.867	0.085	0.781
0.925	0.922	0.879	0.911	0.867	0.097	0.784
0.950	0.925	0.873	0.910	0.866	0.104	0.786
0.975	0.924	0.879	0.911	0.866	0.105	0.786

Table S6. Preliminary external cross-domain validation on three public stomatal datasets without fine-tuning.
mAP50, mAP75, and mAP95: mean Average Precision at IoU thresholds of 0.50, 0.75, and 0.95, respectively; mAP50–95: mean Average Precision averaged over IoU thresholds from 0.50 to 0.95; MAE: mean absolute error; Center F1: F1-score based on one-to-one center matching.

Dataset	Method	mAP50	mAP75	mAP95	mAP50–95	Count MAE	Bias	Center F1
OilPalm/Kwong2021	YOLOv11-OBB	0.507	0.407	0.000	0.324	34.18	-34.18	0.064
OilPalm/Kwong2021	DFA-YOLO	0.633	0.445	0.005	0.403	22.26	-22.26	0.494
LabelStoma/Casado2020	YOLOv11-OBB	0.459	0.376	0.002	0.317	29.76	-26.76	0.276
LabelStoma/Casado2020	DFA-YOLO	0.505	0.386	0.012	0.330	23.04	-3.12	0.409
Toda2021/Wheat imprint	YOLOv11-OBB	0.532	0.344	0.002	0.326	69.55	-69.53	0.180
Toda2021/Wheat imprint	DFA-YOLO	0.550	0.496	0.005	0.394	51.93	-46.15	0.451

Table S7. Five-fold plant-wise cross-validation results of YOLOv11-OBB baseline and DFA-YOLO. Panel A reports mean ± SD across five folds. Panel B reports the fold-wise mAP50 – 95 values of the two models under identical plant-wise partitions and the result of a paired two-sided Wilcoxon signed-rank test. P: Precision; R: Recall; mAP50, mAP75, and mAP95: mean Average Precision at IoU thresholds of 0.50, 0.75, and 0.95, respectively; mAP50 – 95: mean Average Precision averaged over IoU thresholds from 0.50 to 0.95. The paired two-sided Wilcoxon signed-rank test on fold-wise mAP50 – 95 values was not statistically significant ($p = 0.188$).

A. Mean ± SD across five folds

Model	P	R	mAP50	mAP75	mAP95	mAP50–95
YOLOv11-OBB	0.838 ± 0.083	0.918 ± 0.050	0.927 ± 0.049	0.872 ± 0.089	0.005 ± 0.006	0.683 ± 0.080
DFA-YOLO	0.866 ± 0.070	0.926 ± 0.043	0.946 ± 0.039	0.875 ± 0.107	0.010 ± 0.010	0.701 ± 0.094

B. Fold-wise mAP50 – 95 under identical plant-wise partitions

Fold	YOLOv11-OBB mAP50–95	DFA-YOLO mAP50–95	Difference (DFA − baseline)
1	0.751	0.784	0.033
2	0.783	0.808	0.026
3	0.650	0.695	0.044
4	0.597	0.601	0.004
5	0.633	0.620	-0.013

Table S8. Task-aligned comparison between StoManager1 and DFA-YOLO on the original, non-augmented held-out multi-stomata test set. The test set contained 50 maize stomatal microscopic images and 3959 manually annotated stomatal instances. Count-level results were obtained using the dedicated task-aligned export protocol developed for comparison with StoManager1. Conf: confidence threshold; MAE: mean absolute error; RMSE: root mean square error; Pearson r: Pearson correlation coefficient between predicted and manually annotated stomatal counts.

A. Count-level comparison

Method	Setting	Conf	Count MAE	Count RMSE	Count bias	Pearson r
StoManager1	val-calibrated	0.005	28.66	38.06	-17.06	0.509
DFA-YOLO	default	0.12	23.54	26.26	23.54	0.943
DFA-YOLO	val-calibrated	0.60	8.22	10.06	4.50	0.977

B. Center-matching comparison

Method	Setting	Precision	Recall	F1-score
StoManager1	val-calibrated	0.713	0.560	0.627
DFA-YOLO	default	0.768	0.996	0.867
DFA-YOLO	val-calibrated	0.892	0.943	0.917

Table S9. Diagnostic decomposition of the precision–recall trade-off underlying Table 6 on the held-out maize multi-stomata test set. TP, FP, and FN were obtained by one-to-one OBB matching at IoU ≥ 0.5. Pred/image = TP/image + FP/image. Count bias/image = Pred/image − GT/image, where GT/image = 3959/50 = 79.18. Because the density estimate in the present workflow is count-based, count bias per image provides a practical proxy for the effect on stomatal density estimates.

Model	Table 6 P	Table 6 R	TP/image	FP/image	FN/image	Pred/image	Count bias/image
YOLOv5-OBB	0.556	0.516	40.86	32.63	38.32	73.48	-5.70
YOLOv8-OBB	0.925	0.786	62.24	5.05	16.94	67.28	-11.90
YOLOv10-OBB	0.920	0.780	61.76	5.37	17.42	67.13	-12.05
YOLOv11-OBB	0.923	0.786	62.24	5.19	16.94	67.43	-11.75
DFA-YOLO	0.875	0.900	71.26	10.18	7.92	81.44	+2.26

Table S10. Agreement analysis between manual OBB annotations and DFA-YOLO predictions. R^2 was calculated using r2_score(manual annotation, prediction), not Pearson r^2. RMSE: root mean square error; MAE: mean absolute error; Bias: prediction − manual annotation; Bland–Altman 95% LoA: bias ± 1.96 × SD of paired differences. Pearson r and P-value indicate correlation strength and significance. Stomatal count was evaluated per image using the validation-calibrated inference setting, while major and minor axis lengths were calculated from one-to-one matched OBB pairs with IoU ≥ 0.5.

Metric	R^2	RMSE (stomata/pixels)	MAE (stomata/pixels)	Bias	Bland-Altman 95% LoA	Pearson r	P-value	Matched Pairs(images/stomata)
Stomatal Count	0.899	10.06	8.22	4.50	[-13.31, 22.31]	0.977	<0.001	50 images
Major Axis Length	0.869	5.58	4.02	-2.28	[-12.26, 7.69]	0.945	<0.001	3730 stomata
Minor Axis Length	0.873	5.50	4.04	2.75	[-6.60, 12.10]	0.953	<0.001	3730 stomata

Table S11. Comparison of detection-oriented stomatal descriptors between well-watered and water-stressed maize leaves. Values are presented as mean ± SEM (standard error of the mean). WW: well-watered control; WS: water stress (drought). Stomatal area, major axis length, and minor axis length were derived from OBB predictions. Statistical significance was determined by independent samples t-test. *** $P < 0.001$; ns, not significant.

Characteristic	WW (Control)	WS (Drought)	t-statistic	P-value
Stomatal Density (stomata/image)	53.74±1.00	66.30±0.92***	-8.98	<0.001
Stomatal Area (pixels^2)	4163.39±16.55	3751.37±14.59***	18.61	<0.001
Major Axis Length (pixels)	70.67±0.13	70.64±0.14ns	0.14	0.889
Minor Axis Length (pixels)	57.87±0.16	52.21±0.14***	26.92	<0.001
